# Individuality and generality of intratumoral microbiome in the three most prevalent gynecological malignancies: an observational study

**DOI:** 10.1128/spectrum.01004-24

**Published:** 2024-08-05

**Authors:** Qin Xiao, Wen-jie Chen, Fei Wu, Xin-yi Zhang, Xia Li, Jing Wei, Ting-tao Chen, Zhao-xia Liu

**Affiliations:** 1 Departments of Reproductive Medicine, The Second Affiliated Hospital, Jiangxi Medical College, Nanchang University, Nanchang, China; 2 Queen Mary School, Jiangxi Medical College, Nanchang University, Nanchang, China; 3 National Engineering Research Center for Bioengineering Drugs and the Technologies, Institution of Translational Medicine, Jiangxi Medical College, Nanchang University, Nanchang, China; 4 Department of Assisted Reproduction, Maternity and Child Health Hospital of Jiujiang, Jiujiang, Jiangxi, China; 5 School of Pharmacy, Jiangxi Medical College, Nanchang University, Nanchang, China; Foundation for Innovative New Diagnostics, Geneve, Switzerland

**Keywords:** intratumoral microbiome, tumor bacteria, 16S amplicon high throughput sequencing, cervical cancer, ovarian cancer, endometrial cancer

## Abstract

Growing evidence have indicated the crucial role of intratumor microbiome in a variety of solid tumor. However, the intratumoral microbiome in gynecological malignancies is largely unknown. In the present study, a total of 90 *Han* patients, including 30 patients with cancer in cervix, ovary, and endometrium each were enrolled, the composition of intratumoral microbiome was assessed by 16S rDNA amplicon high throughput sequencing. We found that the diversity and metabolic potential of intratumoral microbiome in all three cancer types were very similar. Furthermore, all three cancer types shared a few taxa that collectively take up high relative abundance and positive rate, including *Pseudomonas sp*., Comamonadaceae *gen. sp*., *Bradyrhizobium sp*., *Saccharomonospora sp*., *Cutibacterium acnes*, *Rubrobacter sp*., *Dialister micraerophilus*, and *Escherichia coli*. Additionally, *Haemophilus parainfluenzae* and *Paracoccus sp*. in cervical cancer, *Pelomonas sp*. in ovarian cancer, and *Enterococcus faecalis* in endometrial cancer were identified by LDA to be a representative bacterial strain. In addition, in cervical cancer patients, alpha-fetoprotein (AFP) (correlation coefficient = −0.3714) was negatively correlated (*r* = 0.4, 95% CI: 0.03 to 0.7) with *Rubrobacter sp*. and CA199 (correlation coefficient = 0.3955) was positively associated (*r* = 0.4, 95% CI: 0.03 to 0.7) with *Saccharomonospora sp*.. In ovarian cancer patients, CA125 (correlation coefficient = −0.4451) was negatively correlated (*r* = −0.4, 95% CI: −0.7 to −0.09) with *Porphyromonas sp.*. In endometrial cancer patients, CEA (correlation coefficient = −0.3868) was negatively correlated (*r* = −0.4, 95% CI: −0.7 to −0.02) with *Cutibacterium acnes*. This study promoted our understanding of the intratumoral microbiome in gynecological malignancies.

**IMPORTANCE:**

In this study, we found the compositional spectrum of tumor microbes among gynecological malignancies were largely similar by sharing a few taxa and differentiated by substantial species owned uniquely. Certain species, mostly unreported, were identified to be associated with clinical characteristics. This study prompted our understanding of gynecological malignancies and offered evidence for tumor microbes affecting tumor biology among cancers in the female reproductive system.

## INTRODUCTION

Gynecological malignancies are a group of malignant tumors that occur in the female reproductive system and have long become a serious public health problem affecting women health (1). The most common gynecological tumors are mainly seen in *cervix uteri*, *corpus uteri*, and ovary, accounting collectively for 14.4% of all new female cancer cases globally in both 2018 and 2020 and shown a younger tendency (2, 3). Despite the continuous development of clinical methods in the prevention and treatment of gynecological cancers have achieved great success in enhancing prognosis of patients, their pathogenesis and limitation in treatment are still vaguely known. Over 16% new cancer cases are attributable to infectious factors (4). Microbiological agents have also been shown to play a crucial role in tumor biological manifestations and the tumor microenvironment (5–7). Recent studies have indicated that intratumoral microbiome could be a putative target for treatment (8, 9). Consequently, revealing the composition and potential function of intratumoral microbes could be of great significance to develop novel cancer therapeutics.

Systemic report of the extensive existence of intratumor bacteria in various solid tumors has vacillated and subverted the concept that certain tissue in biont is free of germ (10). Further investigation has indicated that tumor bacteria is a promising biomarker in tumor prevention, diagnosis, treatment, and prognosis (5, 6, 11). The effect of tumor bacteria on tumor biology is mainly mediated by microbe-derived metabolites. It is found that these metabolites could affect tumor metabolism, tumor response to radiation, and anti-tumor immunity (9, 12–15). Certain molecules produced by microbes have also shown genotoxicity which may induce or promote tumorigenesis (16–18). Additionally, the tumor bacteria seemed to have crosstalk to the commensal bacteria in the biont, indicating that the tumor bacteria form a large microbiological meshwork to the commensal microbiota (19, 20). Though pan-cancer analysis has drawn an atlas of the diverse microbiome composition in multiple tumor types, bacterial spectrum in gynecological malignancies remained largely unknown (21, 22).

The female reproductive system shares a uniformed developmental ontogeny and has numerous opportunities to be exposed to microorganisms from the open terminus, particularly vagina and uterus that have even welcomed residents of beneficial microbes like *Lactobacillus* from long-term convergent evolution (23–25). The current data indicated that the intratumoral microbiome of gynecological malignancies is deeply correlated with vaginal microbiome, and vaginal dysbiosis could be a risk factor in various gynecological diseases, including tumors as well (26–28). Growing evidence have suggested that intratumoral microbiome could potentiate tumoral progression. Sheng et al. (29) reported five strains of bacteria including *Achromobacter deleyi Microcella alkaliphila*, *Devosia sp*. LEGU1, *Ancylobacter pratisalsi*, and *Acinetobacter seifertii* are strongly associated with M_1_-polarized macrophage in tumor microenvironment. Jiang et al. (30) reported six gena of bacteria including *Robiginitomaculum*, *Klebsiella*, *Micromonospora*, *Microbispora*, and *Methylobacter* associated with metastasis of cervical cancer. Huang et al. (31) reported that *Propionibacterium acnes* isolated from epithelial ovarian cancer patients promotes ovarian cancer progression in mice models via Hedgehog signaling. However, the shared bacterial taxa among cancer types in the female reproductive system are seldom reported and discussed. Consequently, identification of the mutual intratumoral microbial community could be of great significance to understanding the effects of tumor bacteria on cancer hallmarks.

Herein, we designed an observational study to investigate the difference and generality of intratumoral microbiome among gynecological cancer in cervix, endometrium, and ovary. The clinical data of patients are recorded. The 16S rRNA high-throughput sequencing was conducted for analysis of intratumoral microbiome. The correlation between the diversity of bacteria in tumor and clinical biochemical indicators and tumor markers was analyed. The differential composition of intratumoral microbiome between tumors exposed to the external environment or not is discussed. This study aims to provide information for further microbiome study in gynecological malignancies and offer insight into the prevention and treatment of gynecological tumor in the future.

## MATERIALS AND METHODS

### Object of study

A total of 90 patients, hospitalized in the Department of Obstetrics and Gynaecology of Second Affiliated Hospital of Nanchang University from March 2019 to December 2021, were included and divided into three groups by cancer types. Patients were included if the patient meets all of the following criteria: (1) aged between 18 and 70; (2) pathologically diagnosed with ovary, cervix, or endometrium cancer and accepted for surgery; (3) no antibiotics used for the resent 3 months; (4) generally healthy and no underlying condition were identified; and (5) willing to participate and sign consent forms. Patients were excluded if the patient meets one of the following criteria: (1) severe heart, lung, kidney, and liver dysfunction, or metabolic disease(s); (2) other disease(s) found at the primary site of the tumor; (3) previous cancer history; or (4) two or more malignant tumors presented simultaneously. Three groups are named as (1) Cervical cancer group (Cervix), composed of 30 patients with cervical cancer (*n* = 30); (2). Ovarian cancer group (Ovary), composed of 30 patients with ovarian cancer (*n* = 30); and (3) Endometrial cancer group (Endometrium), composed of 30 patients with endometrial cancer (*n* = 30).

### Sample collection

To acquire a tumor sample, the surface of the collected tumor was immediately disinfected with iodophor after surgical resection. The inner tissue was incised and transferred to a 1.5 mL centrifuge tube containing 1 mL of 50% glycerol-water (vol/vol) solution. All instruments and reagents were strictly sterilized to avoid contamination. All tumor samples were stored at −80°C. The tumor sampling was conducted with the non-blind method.

### DNA extraction, amplification, and 16S high-throughput amplicon sequencing

The genomic DNA in tumor samples was extracted with a bacterial DNA extraction kit (DP302, TIANGEN Biotech). The rDNA of bacterial ribosome subunit 16S (V1-V9 region) was amplified using universal primer 27F and 1492R (32). As described earlier, after compiling, quality inspection, quantification, and proportional mixing, the 16S-encoding DNA samples were sequenced using the PacBio Sequel II platform (Supplementary Information S2) (33). The bacterial DNA extraction and 16S rRNA high-throughput amplicon sequencing were conducted with the double-blind method.

### Bioinformatical processing of raw data in sequencing

The sequenced raw data were first processed by depriming, mass filtering, denoise, splicing, dechimerism, and cluster analysis using Vsearch. In detail, the primer fragment of the sequence was cut by cutadapt module, set −0 to 10, and discard the sequence of unmatched primers. Sequences were concatenated using fastq mergepairs module. The quality of the splicing sequences was controlled by fastq filter module. The duplicate sequences were removed by derep fulllength module. The de-duplicated sequences were clustered at 98% similarity level by the cluster size module, and the chimeras were removed by uchime *de novo* module. To obtain high-quality sequence, perl script (https://github.com/torognes/vsearch/wiki/VSEARCH-pipeline) was used to filter quality control sequence concentrated chimeras.

The high-quality sequences were clustered at 97% similarity level and output representative sequences and operational taxonomic unit (OUT) tables by cluster size module, respectively. To reduce accidental error, singletons OTUs, namely OTUs with an abundance of 1 in all samples, and their representative sequences were removed from the OTU table. The α diversity indices, including Chao 1, Observed species, and Faith’s phylogenetic diversity (Faith’s PD), and β diversity, including principal coordinate analysis (PCoA) were analyed. The relative abundance of bacterial taxa is shown in percentage (%).

To annotate the taxonomic classification of sequence, NCBI (https://ftp.ncbi.nih.gov/blast/db/) was used as a reference sequence database. For identifying representative bacterial strain, linear discriminant analysis (LDA) (34), LDA Effect Size (LEfSe) analysis (35), and Random Forests analysis were conducted. For LDA and LEfSe analyses, power comparison control strategy was set as one-against-one, LDA threshold was set at 4, and classification level threshold was set at 0.05. The Random Forests analysis was performed by invoking classify_samples_ncv function of q2-sample-classifier to conduct analysis using unrarefied ASV/OUT table-generated absolute taxonomic abundance table, with nested hierarchical cross-validations (10-fold cross-validations).

### Statistical analysis

Statistical analysis of this study was performed by Prism (10.1.1, GraphPad, USA). Numerical data are presented as mean ± SD. To perform the analysis, data were first tested for normal distribution by Shapiro-Wilk test. For data not normally distributed, statistical significance was determined by Kruskal-Wallis test followed by Dunn’s multiple comparison. For data normally distributed, statistical significance was determined by one-way ANOVA followed by Turkey’s multiple comparison. In correlation analysis, the correlation coefficient was computed by Pearson analysis if both data sets were normally distributed, otherwise correlation coefficient was computed by nonparametric Spearman analysis, all of which follows two-tailed statistical significance test, and confidence interval (CI) was set at 95%. *: *P* < 0.05, **: *P* < 0.01, and ***: *P* < 0.001.

## RESULTS

### Microbiome diversity among cancers in different sites

**Fig 1:**
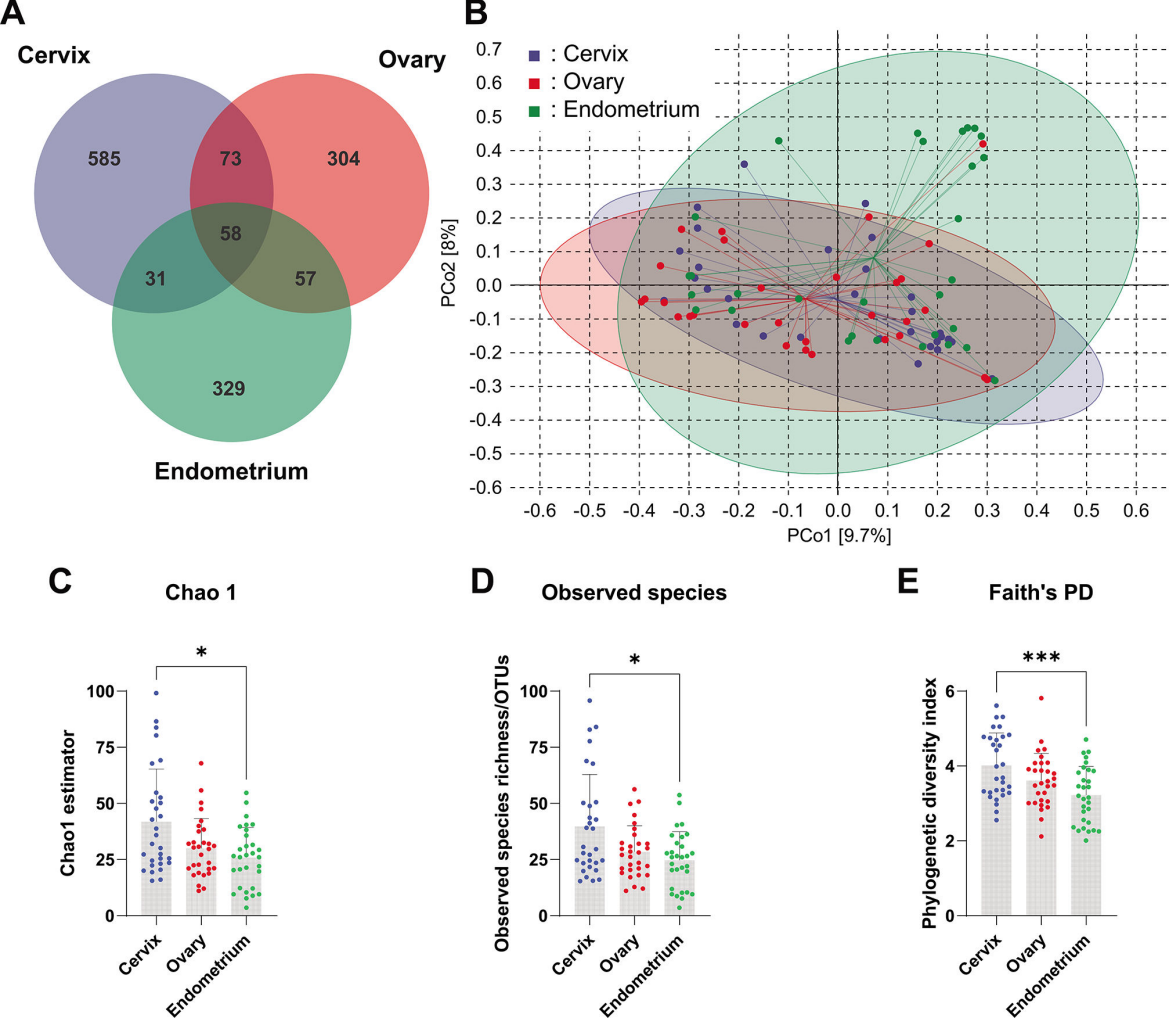
Microbiome diversity among cancers in cervix, ovary, and endometrium. (A). Venn diagram showing numbers of exclusive and shared OTUs among three groups. (B). Distance matrices showing principal coordinates analysis (PCoA) PCo1 (*X* axis) and PCo2 (*Y* axis) data. C–E. Statistical analysis of α indices including Chao1 (C), Observed species (D) ,and Faith’s phylogenetic diversity (PD) (E) among three groups. For numerical analyses, statistical significance was obtained by Kruskal-Wallis test following Dun’s multiple comparison (C and D) or one-way ANOVA following Turkey’s multiple comparison (E). *: *P* < 0.05 and ***: *P* < 0.001. For distance matrices, the distance algorithm was set as Bray–Curtis, and the elliptic confidence was set at 95%.

As shown in Fig. 1A, a total of 1,437 operational taxonomic units (OTUs) were identified; 585, 304, and 329 OTUs were found exclusively from cancer in cervix, ovary, and endometrium, respectively, and 73 OTUs shared by cervix and ovary cancers, 57 OTUs shared by ovary and endometrium cancer, 31 OTUs shared by endometrium and cervix cancer, and 58 OTUs shared by all three cancer types.

The principal coordinate analysis (PCoA) (Fig. 1B) suggested that there was a trivial separation among the three groups. Although certain samples were localied outside of the confidence ellipse, the distance of samples was similar and consistent with the grouping. Furthermore, no evident distortion of overlapping confidence ellipse was observed. The prediction of overall metabolic activity of intratumoral microbiome also showed a similar manner in cancer biology (Supplementary information S3), indicating that the putative metabolic function of intratumoral function is shared among cancers in cervix, ovary, and endometrium.

For α diversity, three indices, including Chao 1 (Cervix vs. Ovary vs. Endometrium = 41.91 ± 23.35 vs. 29.97 ± 13.29 vs. 25.89 ± 13.30), Observed species (Cervix vs. Ovary vs. Endometrium = 39.79 ± 23.07 vs. 28.43 ± 11.61 vs. 24.74 ± 12.61), and Faith’s PD (Cervix vs. Ovary vs. Endometrium = 4.016 ± 0.8661 vs. 3.614 ± 0.7223 vs. 3.223 ± 0.7649) were selected (Fig. 1C through E). The results showed that the cervix cancer patients have much higher microbial diversity than endometrium cancer patients in Chao 1 (*P* < 0.05), observed species (*P* < 0.05), and Faith’s PD (*P* < 0.01).

These findings indicated that the microbiome composition is largely similar by sharing a few bacterial taxa among cancers in cervix, ovary, and endometrium, while also a large number of bacterial taxa existed distinctively among all three cancers to compile a small portion of the intratumoral microbiome.

### Taxonomic composition of intratumoral microbiome in patients

**Fig 2:**
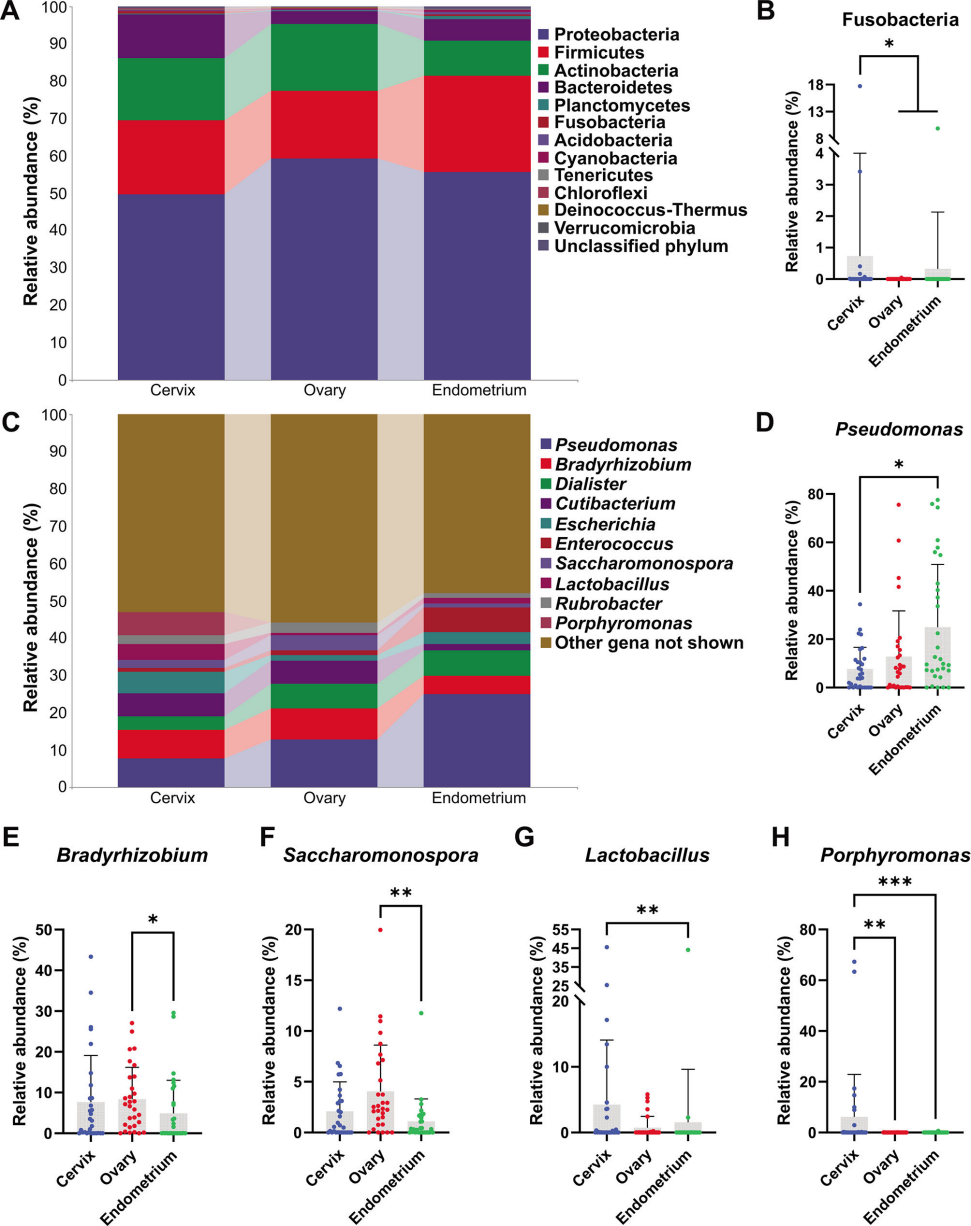
Taxonomic composition of intratumoral microbiome in patients. A. Taxonomic column diagram at phylum level showing twelve identified phyla and one unclassified phylum. B. Statistical analysis of phylum Fusobacteria among three cancer types. C. Taxonomic column diagram at genus level showing overall top ten most abundant identified gena, all other gena are merged as other. D-H. Statistical analysis of genus *Pseudomonas* (D), *Bradyrhizobium* (E), *Saccharomonospora* (F), *Lactobacillus* (G), and *Porphyromonas* (H) among three cancer types. Statistical significances were all obtained with Kruskal-Wallis test followed by Dunn’s multiple comparison. *: *P* < 0.05, **: *P* < 0.01, and ***: *P* < 0.001.

To explore the primary component of intratumoral microbiome, the composition and relative abundance of intratumoral microbiome among three cancers were analyed, as shown in Fig. 2. At phylum, class, order, family, genus, and species level, a total of 13, 35, 68, 131, 237, and 299 taxa (including unclassified taxa) were identified (Supplementary Information S1).

At the phylum level, all phyla were analyed (Fig. 2A). The phylum Fusobacteria (Cervix vs. Ovary vs. Endometrium = 0.7266 ± 3.269 vs. 0.001447 ± 0.007924 vs. 0.3287 ± 1.801) in cervix cancer patients was found to be increased than ovary (*P* < 0.05) and endometrium (*P* < 0.05) cancer patients (Fig. 2B). No other phylum was found to be statistically enriched among the three cancer types.

The top 10 most abundant gena, which take up 44.14–52.12% of the total microbiome, were analyed (Fig. 2C). Genus *Pseudomonas* (Cervix vs. Ovary vs. Endometrium = 7.726 ± 8.877 vs. 12.81 ± 18.92 vs. 25.00 ± 25.88) were found to be markedly increased in endometrial cancer compared to the cervical cancer (*P* < 0.05) (Fig. 2D). Genus *Bradyrhizobium* (Cervix vs. Ovary vs. Endometrium = 7.635 ± 11.45 vs. 8.343 ± 7.857 vs. 4.885 ± 8.121) and genus *Saccharomonospora* (Cervix vs. Ovary vs. Endometrium = 2.092 ± 2.906 vs. 4.056 ± 4.546 vs. 10.91 ± 2.220) were found to be markedly increased in ovarian cancer compared with the endometrial cancer (*P* < 0.05 and *P* < 0.01, respectively) (Fig. 2E and F). Genus *Lactobacillus* (Cervix vs. Ovary vs. Endometrium = 4.224 ± 9.847 vs. 0.7442 ± 1.725 vs. 1.550 ± 8.060) were found to be markedly increased in cervical cancer compared with the endometrial cancer (*P* < 0.01) (Fig. 2G). Genus *Porphyromonas* (Cervix vs. Ovary vs. Endometrium = 6.178 ± 16.71 vs. 0.008149 ± 0.02107 vs. 0.02117 ± 0.1121) was found to be markedly increased in cervical cancer compared with ovarian cancer (*P* < 0.01) and endometrial cancer (*P* < 0.001) (Fig. 2H).

### Screening gynecological malignancies-shared bacterial strain

**Fig 3:**
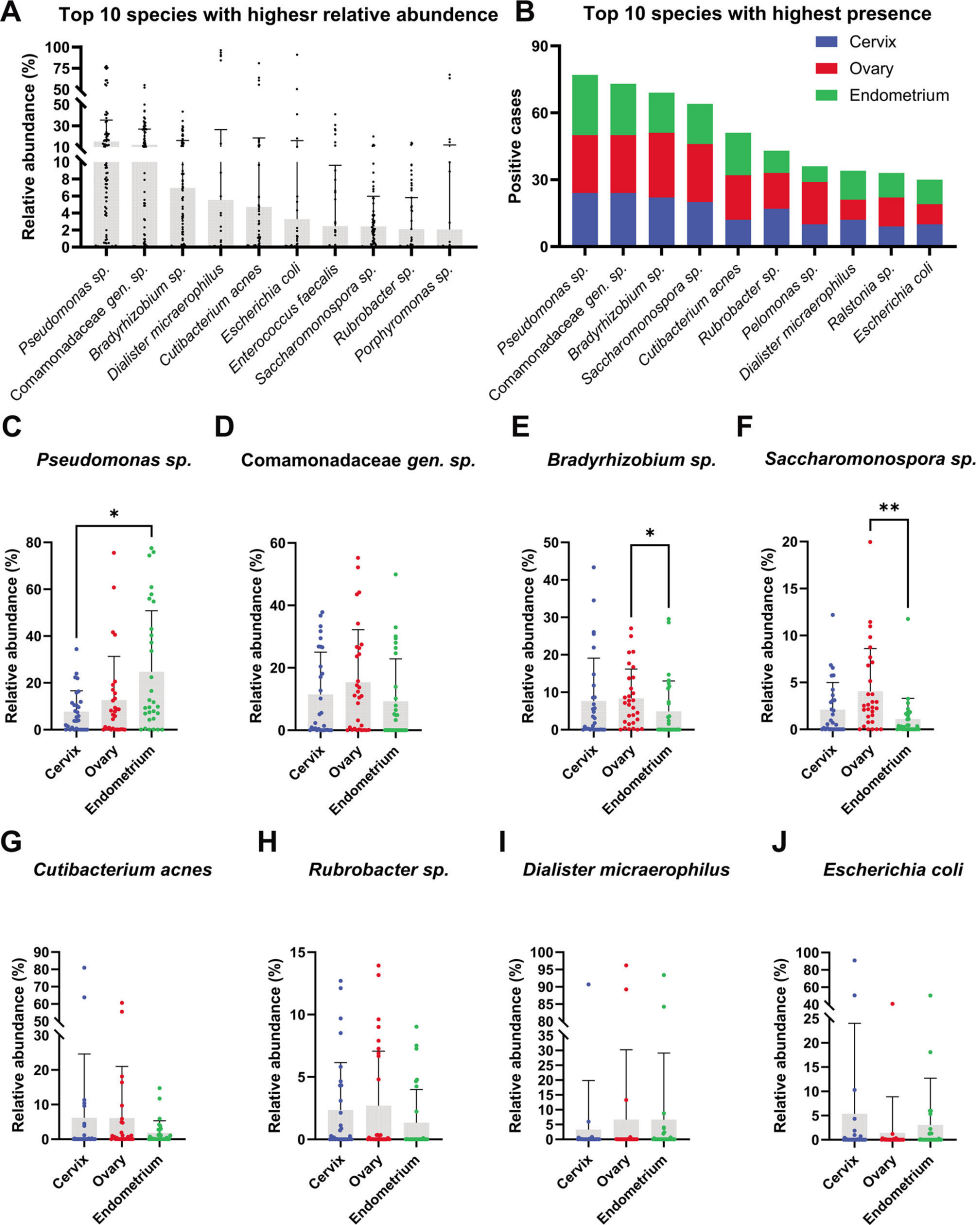
Common bacterial species among gynecological malignancies. A-B. Histogram showing the top 10 species with highest relative abundance (A) and positive ratio (B). C-J. Statistical analysis of intersected species from the top 10 species, including *Pseudomonas sp*., Comamonadaceae *gen. sp*., *Bradyrhizobium sp*., *Saccharomonospora sp*., *Cutibacterium acnes*, *Rubrobacter sp*., *Dialister micraerophilus*, and *Escherichia coli*, with the highest relative abundance and positive ratio. Statistical significance was assessed using Kruskal-Wallis test following Dunn’s multiple comparison. *: *P* < 0.05 and **: *P* < 0.01.

The aforementioned findings have shown that a few numbers of taxa shared among three gynecological malignancies are the main contributors to the intratumoral microbiome. To identify these chassis strains, the bacterial species were sorted by overall relative abundance and overall detection ratio, then take the intersection set from the top 10 of them, as shown in Fig. 3. The results showed that eight species, including *Pseudomonas sp*. (found in 77 out of 90 cases with relative abundance of 15.05 ± 20.31), Comamonadaceae *gen. sp*. (found in 73 out of 90 cases with relative abundance of 12.06 ± 14.78), *Bradyrhizobium sp*. (found in 69 out of 90 cases with relative abundance of 6.954 ± 9.304), *Saccharomonospora sp*. (found in 64 out of 90 cases with relative abundance of 2.413 ± 3.553), *Cutibacterium acnes* (formerly termed *Propionibacterium acnes*) (found in 51 out of 90 cases with relative abundance of 4.721 ± 13.82), *Rubrobacter sp*. (found in 43 out of 90 cases with relative abundance of 2.129 ± 3.683), *Dialister micraerophilus* (found in 34 out of 90 cases with relative abundance of 5.536 ± 20.89), and *Escherichia coli* (found in 30 out of 90 cases with relative abundance of 3.283 ± 12.81), were found in the intersection set (Fig. 3A and B).

In addition, it is found that the relative abundance of *Pseudomonas sp*. (Cervix vs. Ovary vs. Endometrium = 7.726 ± 8.877 vs. 12.65 ± 18.66 vs. 24.75 ± 26.08) was markedly increased in endometrial cancer than in cervical cancer (*P* < 0.05) (Fig. 3C), and relative abundance of *Bradyrhizobium sp*. (Cervix vs. Ovary vs. Endometrium = 7.635 ± 11.45 vs. 8.343 ± 7.857 vs. 4.885 ± 8.121) and *Saccharomonospora sp*. (Cervix vs. Ovary vs. Endometrium = 2.092 ± 2.906 vs. 4.056 ± 4.546 vs. 1.091 ± 2.220) were markedly increased in ovarian cancer than in endometrial cancer (*P* < 0.05 and *P* < 0.01, respectively) (Fig. 3E and F).

### Screening gynecological malignancies-representative bacterial strain

**Fig 4:**
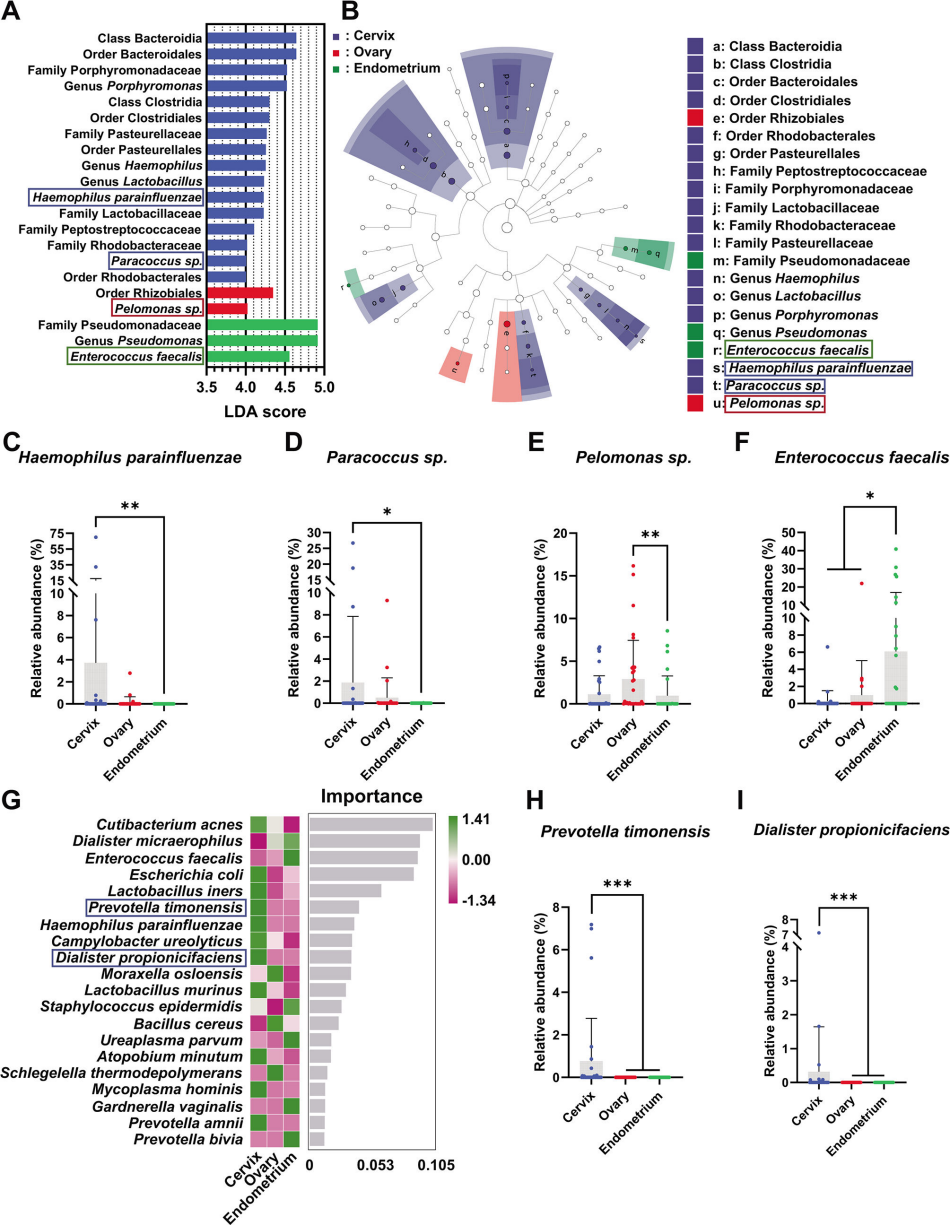
Distinctive bacterial species among gynecological malignancies. A. Histogram of LDA score among taxa, showing taxa with LDA score ≥4. B. Taxonomic cladistics of LDA Effect Size (LEfSe) analysis. C-F. Statistical analysis of bacterial strains, including *Haemophilus parainfluenzae* (C), *Paracoccus sp*. (D), *Pelomonas sp*. (E), and *Enterococcus faecalis* (F), with LDA score ≥4. G. Random Forest analysis of the intratumoral microbiome at the species level. H-I. Statistical analysis of bacterial strains, including *Prevotella timonensis* (H) and *Dialister propionicifaciens* (I). For LEfSe analysis, power comparison control strategy was set as one-against-one, LDA threshold was set at 4, and classification level threshold was set at 0.05. For Random Forest analysis and numerical data analysis, statistical significance was obtained using Kruskal-Wallis test following Dun’s multiple comparison. *: *P* < 0.05 and **: *P* < 0.01.

Identification of the shared and uniquely owned microbial taxa among three cancers was further conducted, as shown in Fig. 4. Four distinctive bacterial strains, including *Haemophilus parainfluenzae* (LDA score = 4.229), *Paracoccus sp*. (LDA score = 4.007), *Pelomonas sp*. (LDA score = 4.021), and *Enterococcus faecalis* (LDA score = 4.556), were identified using linear discriminant analysis (LDA) (Fig. 4A). The LEfSe analysis also showed that these bacterial species were cancer type-specific strains (Fig. 4B). Furthermore, it is found that the relative abundance of *Haemophilus parainfluenzae* (Cervix vs. Ovary vs. Endometrium = 3.719 ± 13.89 vs. 0.1270 ± 0.5249 vs. 0.000 ± 0.000) and *Paracoccus sp*. (Cervix vs. Ovary vs. Endometrium = 1.866 ± 5.987 vs. 0.4934 ± 0.1798 vs. 0.001057 ± 0.005790) were significantly higher in cervical cancer compared to endometrial cancer (*P* < 0.01 and *P* < 0.05, respectively) (Fig. 4C and D), while *Pelomonas sp*. (Cervix vs. Ovary vs. Endometrium = 1.127 ± 2.156 vs. 2.903 ± 4.543 vs. 0.9524 ± 2.314) was found to be enriched in ovarian cancer than endometrial cancer (*P* < 0.05) (Fig. 4E) and *Enterococcus faecalis* (Cervix vs. Ovary vs. Endometrium = 0.2850 ± 1.225 vs. 0.9886 ± 4.035 vs. 6.090 ± 10.91) were found to have higher abundance in endometrial cancer than cervical cancer (*P* < 0.05) and ovarian cancer (*P* < 0.05) (Fig. 4F).

In addition, the top 20 most important species were identified by Random Forest analysis (Fig. 4G). Notably, *Cutibacterium acnes* and *Dialister micraerophilus* were the shared species among the three cancer types, while *Enterococcus faecalis* and *Haemophilus parainfluenzae* were the representative species found in endometrial cancer and cervical cancer patients, respectively. It is also noticed that two species, including *Prevotella timonensis* (10 out of 30 patients) and *Dialister propionicifaciens* (7 out of 30 patients), were solely found in a fraction of cervical cancer patients (Fig. 4H and I).

### Correlation between intratumoral microbes and clinical manifestation

**Fig 5:**
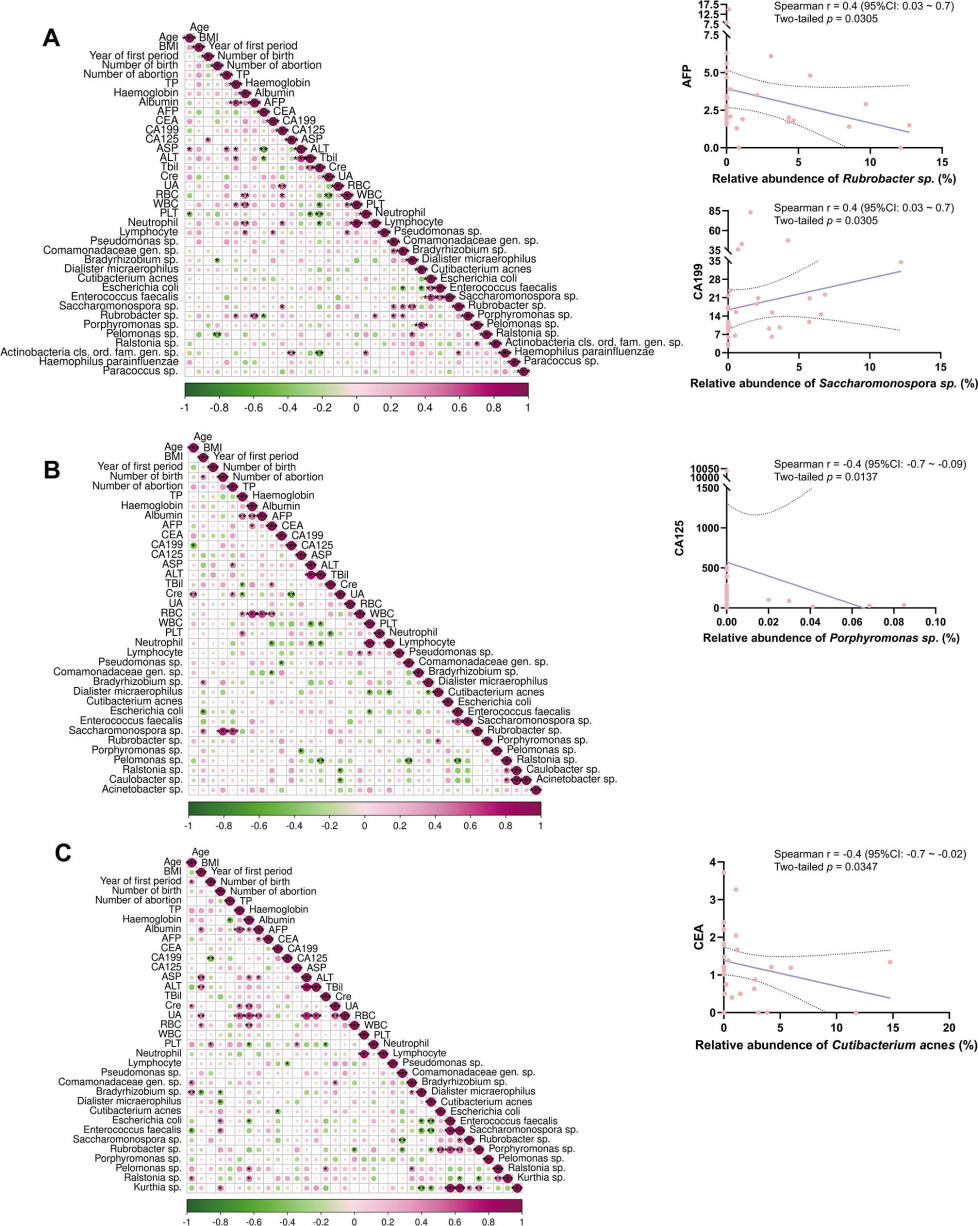
Correlation between intratumoral microbes and clinical manifestation. A–C: Spearman correlation matrix between clinical characteristics and selected species of intratumoral microbiome among patients with cervical (**A**), ovarian (**B**), and endometrial (**C**) cancer, the scatter dot plots highlighted certain correlation analyses. Two-tailed statistical significance was tested, confidence interval (CI) was set at 95%. *: *P* < 0.05, **: *P* < 0.01, and ***: *P* < 0.001.

To further investigate the possible correlation between the intratumoral microbes that have been formerly screened and clinical manifestations, correlation analyses were performed (Fig. 5). The Sperman correlation matrix (Fig. 5) showed the correlation coefficient and statistical significance between clinical characteristics and relative abundance of selected species formerly in intratumoral microbiome in general. Some selected correlations concerning tumor antigen and selected species of intratumoral microbiome were identified and specified. In cervical cancer patients, AFP (correlation coefficient = −0.3714) were found to be negatively correlated (*r* = 0.4, 95% CI: 0.03 to 0.7) with relative abundance of *Rubrobacter sp*. (*P* < 0.05) and CA199 (correlation coefficient = 0.3955) were found to be positively correlated (*r* = 0.4, 95% CI: 0.03 to 0.7) with relative abundance of *Saccharomonospora sp*. (two-tailed *P* < 0.05) (Fig. 5A). In ovarian cancer patients, CA125 (correlation coefficient = −0.4451) were found to be negatively correlated (*r* = −0.4, 95% CI: −0.7 to −0.09) with relative abundance of *Porphyromonas sp*. (two-tailed *P* < 0.05) (Fig. 5B). In endometrial cancer patients, CEA (correlation coefficient = −0.3868) were found to be negatively correlated (*r* = −0.4, 95% CI: −0.7 to −0.02) with relative abundance of *Cutibacterium acnes* (two-tailed *P* < 0.05) (Fig. 5C).

## DISCUSSION

Recent studies have revealed that tumor microbe has ubiquitous distribution in tumor microenvironment and plays non-negligible role in cancer biology (6, 10, 36). Though it has been found that certain bacterial taxa could affect tumor characteristics in gynecological malignancies, it is yet ignorant of the composition of tumor microbes in a systemic way in the female reproductive system. Cervical, endometrial, and ovarian cancers are not only the most common gynecological malignancies but also among the top 10 tumors in terms of the number of new cases of cancer in females (37). Here, the spectrum of intratumoral microbiome in the three most common malignancies, including cervix, ovary, and endometrium, was revealed and compared with 16S high throughput sequencing. The female reproductive system shares a uniformed developmental origination. Ontogeny of cervix, uterine corpus, and ovary are all derived from the genital ridge-differentiated Müllerian duct, Wolffian (mesonephric) duct, and primordial germ cell, which are generated from the nephrogenic cord of mesoderm during the 3rd week of pregnancy (23). However, the commonness among cancers in female reproductive system remained largely unrecognied despite the fact that shared characteristics in pan-cancer research are long and widely discussed (38, 39). Herein, we found that the composition of intratumoral microbiome among cancers in cervix, ovary, and endometrium is surprisingly alike. The OTU numbers among cancers in cervix, ovary, and endometrium showed an anatomically decreasing tendency, namely the lower urogenital tract has more OTUs while the upper urogenital tract has less OTUs, which is coordinated with experience (Fig. 1A). However, this tendency somehow disappeared concerning bacterial diversity indices (Fig. 1C through E). At the phylum level, only Fusobacteria were found to have increased cervical cancer while the composition was much more differentiated at the genus level (Fig. 2). It should be noted that the genus *Lactobacillus* appears to maintain a high abundance in cervical cancer tissue (4.224 ± 9.847), suggesting its precancerous rationale to be drastically different from traditional probiotics. Further analysis at species level, a few strains, including *Pseudomonas sp.*, Comamonadaceae *gen. sp.*, *Bradyrhizobium sp.*, *Saccharomonospora sp.*, *Cutibacterium acnes*, *Rubrobacter sp.*, *Dialister micraerophilus*, and *Escherichia coli*, were found to be the foundation microbes shared by the three cancer types, and all of which has a high abundance and positive rate (Fig. 3). These screened strains were largely unstudied among all three types of cancer. Another bioinformatic study found that genus *Pseudomonas* has a high correlation with high-risk human papilloma virus and cervical cancer in Chinese women, which confirmed our findings (40). More importantly, most of these uncultured bacterial strains are taxonomically classified ambiguously into species level (Supplementary Information S4). The underlying effect on gynecological malignancies and phylogenetic study of these selected chassis bacterial strains depends on further study.

In addition to the shared species, it is of notice that the cancer in cervix, ovary, or endometrium contains many more bacterial species differentiated from the other two types of cancer. The LDA, LEfSe analysis, and Random Forest analysis were conducted to identify disease-specific bacterial species among the three types of cancer (Fig. 4). The LDA and LEfSe analyses showed that four species, including *H, parainfluenzae* and *Paracoccus sp.* in cervical cancer, *Pelomonas sp.* in ovarian cancer, and *E.s faecalis* in endometrial cancer were identified as biomarker species. Studies of *H. parainfluenzae* on cervical cancer remained in bioinformatical study (41). The effect of *Paracoccus sp.* and *Pelomonas sp.* remained untapped. A more well-studied strain is *E. faecalis*, which is elevated in patients with chronic endometritis (42). Zhang et al (43) reported that *E. faecalis* OG1RF could impede endometrial receptivity by superoxide-reliant inflammation, which is also a putative carcinogen in endometrial cancer (44). The effect and underlying mechanisms of disease-specific bacterial species among the three types of cancer require further study. In Random Forest analysis, two species, termed *Prevotella timonensis* (detected in 10 out of 30 patients) and *Dialister propionicifaciens* (detected in 7 out of 30 patients), were found exclusively in certain cervical cancer patients. A bioinformatical study on cervical intraepithelial neoplasia (CIN) also found vaginal *Prevotella timonensis* was associated with CIN2 persistence and slower regression.

To provide insight to further study, we performed correlation analysis (Fig. 5). In cervical cancer patients, AFP was found to be negatively correlated with relative abundance of *Rubrobacter sp.* (*P* < 0.05), and CA199 was found to be positively correlated with relative abundance of *Saccharomonospora sp.* (*P* < 0.05). In ovarian cancer patients, CA125 were found to be negatively correlated with relative abundance of *Porphyromonas sp.* (*P* < 0.05). In endometrial cancer patients, CEA (correlation coefficient = −0.3868) was found to be negatively correlated with relative abundance of *C. acnes* (*P* < 0.05). Among them, the genus *Porphyromonas* is one of the representative microbial taxa of intratumoral microbiome in ovarian cancer (45). Our findings suggested that *Porphyromonas sp.* may have a positive effect on pathophysiology of ovarian cancer. Interestingly, however, Walther-António et al identified an uncultured *Porphyromonas sp.* with 99% similarity to *P. somerae* in the uterine microbiome to be associated statistically with endometrial cancer, particularly with a vaginal pH >4.5 (46). Consequently, the genus *Porphyromonas* may have a dual effect on the pathogenesis of ovarian cancer, which needs to be studied in the future. Chintalapati et al. found that *C. acnes* (family Propionibacteriaceae) isolated from tumor could suppress tumor growth by promoting anti-tumor immunity (47). The effect of *Rubrobacter sp.* and *Saccharomonospora sp.* on tumors remained indefinite.

Collectively, this study shows that the intratumoral microbiome showed a similar composition, and certain species seemed to show a significant role in clinical manifestations in cancer patients. Nevertheless, the findings in this study need to be verified in a larger-scale study to draw a better statistical conclusion. We hope this will further promote studies of the interactions between tumors and the tumor microbiome to provide a new therapeutic strategy.

## Data Availability

The 16S sequencing data are available at BioProject PRJNA1090286 in Sequence Read Archive (SRA) of the National Centre for Biotechnology Information (NCBI). Other data analyzed in the present study are enclosed in the supplementary information.

## SUPPLEMENTAL MATERIAL

Supplemental material spectrum.01004-24-s0001.docx Supplemental methods, tables, and figures.

ASM does not own the copyrights to Supplemental Material that may be linked to, or accessed through, an article. The authors have granted ASM a non-exclusive, world-wide license to publish the Supplemental Material files. Please contact the corresponding author directly for reuse.
