# Supplementary material for: Individuality and generality of intratumoral microbiome in the three most prevalent gynecological malignancies: an observational study
Source: Microbiol Spectr. 2024 Aug 5;12(9):e01004-24. doi: 10.1128/spectrum.01004-24 (PMC11370256; doi:10.1128/spectrum.01004-24)
Supplement: Supplemental material — Supplemental methods, tables, and figures. [file spectrum.01004-24-s0001.docx]

Supplementary Information

**Individuality and Generality of Intratumoural Microbiome in the Three Most Prevalent Gynaecological Malignancies: An Observational Study**

Qin Xiao^a†^, Wen-jie Chen^b,c†^, Fei Wu^a†^, Xin-yi Zhang^b^, Xia Li^d^, Jing Wei^c^, Ting-tao Chen^a,c,e*^, Zhao-xia Liu^a*^

^a^Departments of Reproductive Medicine, The Second Affiliated Hospital, Jiangxi Medical College, Nanchang University, Nanchang 330006, China

^b^Queen Mary School, Jiangxi Medical College, Nanchang University, Nanchang 330031, China

^c^National Engineering Research Center for Bioengineering Drugs and the Technologies, Institution of Translational Medicine, Jiangxi Medical College, Nanchang University, Nanchang 330031, China

^d^Department of Assisted Reproduction, Maternity and Child Health Hospital of Jiujiang, Jiujiang 332000, China

^e^School of Pharmacy, Jiangxi Medical College, Nanchang University, Nanchang 330031, China.

† All three authors have contributed equally to this work and share the first authorship.

Supplementary Materials and Methods

Sample collection

The blood sample was collected from basilic vein during 8 ± 1 in the morning one day before the surgery, with routinely fasted in the previous night. The serum sample was collected using centrifugation at 1000 × g for 5 min after blood clotting at room temperature for 30 min. The blood sampling was conducted with non-blind method and the serum sample collection was conducted with single-blind method.

To acquire tumour sample, the surface of collected tumour was immediately disinfected with iodophor after surgical resection. The inner tissue was incised and transferred to a 1.5 mL centrifuge tube containing 1 mL of 50% glycerol-water (*V/V*) solution. All instruments and reagents were strictly sterilised to avoid contamination. All tumour samples were stored at -80℃. The tumour sampling was conducted with non-blind method.

Hemogram, blood biochemistry, and tumour biomarker

The hemogram and blood biochemistry was tested by professional laboratorians using automatic haemocytometer analyser (XE-2100, Sysmex, Japan) and automatic biochemical analyser (CX7, Beckman, USA), respectively. The tumour biomarkers, including α-fetoprotein (AFP), carcino-embryonic antigen (CEA), squamous cell carcinoma antigen (SCCA), carbohydrate antigen (CA) 199, and CA125, were tested by professional laboratorians using chemiluminescent immunoassay (CLIA) with automatic chemiluminescence apparatus (Cobas E411, Roche, Germany). The minimum detection limit of AFP and CEA is 1.3 ng/mL and 0.5 ng/mL, respectively. For serum level lower than detection limit is recorded as 0 ng/mL. The blood tests were conducted with double-blind method.

Functional prediction of intratumoural microbiome

The composition of bacterial community genes or functional units was predicted with reference to the known microbial genomic data using phylogenetic investigation of communities by reconstruction of unobserved states (PICRUSt2) (<https://github.com/picrust/picrust2/wiki>). By default, one millionth of the total abundance of KO and EC for each sample is used, respectively. Metabolic pathway abundance files (i.e. path_abun_unstrat.tsv and path_abun_strat.tsv) and functional unit files (i.e. pred_metagenome_unstrat.tsv and pred_metagenome_strat.tsv) were normalised, so that the abundance value is in units of “per million functional units”. The PCoA analysis based on functional prediction was also performed.

Supplementary Information S1 Patients Clinical Characteristics

Baseline characteristics of patients

| **Variables** | **Cervix**  **(*n* = 30)** | **Ovary**  **(*n* = 30)** | **Endometrium**  **(*n* = 30)** | **P value** |
| --- | --- | --- | --- | --- |
| **Age (yrs.)** | 50.60 ± 10.08 | 51.37 ± 12.06 | 54.03 ± 7.049 | 0.3775 |
| **BMI (kg/m^2^)** | 23.37 ± 2.718 | 23.02 ± 3.543 | 23.36 ± 4.012 | 0.6929 |
| **Menarche age (yrs.)** | 13.67 ± 0.9589 | 13.63 ± 1.066 | 13.87 ± 1.137 | 0.7828 |
| **Number of births** | 2.633 ± 1.098 | 2.167 ± 1.392 | 2.033 ± 0.8087 | 0.0738 |
| **Number of abortions** | 1.300 ± 1.264 | 0.7333 ± 0.9072 | 0.5000 ± 0.9377 | 0.0141^a^ |
| **Cancer type**  Squamous carcinoma  Adenomatous carcinoma  Other | 29  0  1 | 0  25  5 | 0  30  0 | / |
| **Staging**  I  Ⅱ  Ⅲ  Ⅳ | 22  5  2  1 | 8  4  12  6 | 23  2  3  2 | / |
| **Metastasis**  Positive  Negative | 1  29 | 6  24 | 3  27 | / |
| **HPV infection**  High risk  Other | 18  12 | 2  28 | 1  29 | / |

a: *p* < 0.05 between group Cervix and Endometrium

History

| **Patient ID** | **Age (years)** | **BMI (kg/m^2^)** | **Age of menarche (years)** | **Birth** | **Abortion** | **Pathology** |
| --- | --- | --- | --- | --- | --- | --- |
| C01 | 45 | 22.06 | 14 | 1 | 0 | SCC |
| C02 | 55 | 21.09 | 13 | 3 | 0 | AC |
| C03 | 53 | 24.22 | 15 | 2 | 0 | SCC |
| C04 | 64 | 23.63 | 14 | 2 | 0 | SCC |
| C05 | 54 | 24.65 | 12 | 3 | 0 | SCC |
| C06 | 36 | 21.97 | 13 | 2 | 2 | SCC |
| C07 | 50 | 23.95 | 15 | 3 | 1 | SCC |
| C08 | 64 | 26.14 | 13 | 4 | 1 | SCC |
| C09 | 51 | 23.11 | 15 | 4 | 0 | SCC |
| C10 | 45 | 25.39 | 14 | 2 | 0 | SCC |
| C11 | 41 | 24.46 | 13 | 2 | 3 | SCC |
| C12 | 62 | 24.44 | 15 | 3 | 1 | SCC |
| C13 | 49 | 19.96 | 15 | 2 | 2 | SCC |
| C14 | 53 | 20.03 | 13 | 3 | 0 | SCC |
| C15 | 39 | 22.51 | 12 | 2 | 2 | SCC |
| C16 | 61 | 29.14 | 12 | 5 | 3 | SCC |
| C17 | 77 | 23.08 | 12 | 6 | 0 | SCC |
| C18 | 32 | 20.88 | 14 | 4 | 2 | NEC |
| C19 | 49 | 25.78 | 14 | 3 | 2 | SCC |
| C20 | 52 | 22.89 | 14 | 2 | 1 | AC |
| C21 | 63 | 22.89 | 13 | 2 | 2 | SCC |
| C22 | 40 | 19.68 | 14 | 1 | 2 | SCC |
| C23 | 39 | 24.97 | 14 | 2 | 2 | SCC |
| C24 | 34 | 20.03 | 14 | 2 | 4 | SCC |
| C25 | 49 | 18.05 | 13 | 2 | 4 | SCC |
| C26 | 53 | 22.03 | 13 | 3 | 3 | SCC |
| C27 | 53 | 26.99 | 14 | 2 | 1 | SCC |
| C28 | 57 | 22.89 | 14 | 2 | 0 | SCC |
| C29 | 52 | 30.3 | 14 | 3 | 1 | SCC |
| C30 | 46 | 23.91 | 15 | 2 | 0 | SCC |
| E01 | 63 | 20.69 | 12 | 2 | 1 | AC |
| E02 | 57 | 21.97 | 13 | 2 | 2 | AC |
| E03 | 65 | 22.67 | 14 | 3 | 0 | AC |
| E04 | 59 | 24.41 | 14 | 1 | 0 | AC |
| E05 | 54 | 22.38 | 15 | 2 | 1 | AC |
| E06 | 57 | 20.2 | 15 | 3 | 0 | AC |
| E07 | 58 | 24.61 | 15 | 2 | 0 | AC |
| E08 | 47 | 21.09 | 14 | 2 | 2 | AC |
| E09 | 51 | 23.93 | 14 | 2 | 0 | AC |
| E10 | 49 | 31 | 15 | 2 | 1 | AC |
| E11 | 45 | 23.73 | 16 | 2 | 0 | AC |
| E12 | 58 | 21.93 | 12 | 2 | 0 | AC |
| E13 | 48 | 21.4 | 15 | 2 | 0 | AC |
| E14 | 48 | 30.84 | 13 | 2 | 1 | AC |
| E15 | 53 | 25.85 | 14 | 2 | 0 | AC |
| E16 | 64 | 13.68 | 13 | 3 | 0 | AC |
| E17 | 46 | 22.83 | 15 | 3 | 2 | AC |
| E18 | 68 | 23.91 | 13 | 3 | 4 | AC |
| E19 | 64 | 23.24 | 12 | 4 | 0 | AC |
| E20 | 53 | 21.5 | 13 | 1 | 0 | AC |
| E21 | 46 | 26.67 | 13 | 1 | 0 | AC |
| E22 | 46 | 21.51 | 16 | 1 | 0 | AC |
| E23 | 52 | 30.36 | 13 | 0 | 0 | AC |
| E24 | 40 | 23.04 | 13 | 1 | 0 | AC |
| E25 | 48 | 32.44 | 14 | 3 | 1 | AC |
| E26 | 57 | 19.23 | 15 | 2 | 0 | AC |
| E27 | 62 | 17.31 | 13 | 2 | 0 | AC |
| E28 | 56 | 25.02 | 15 | 2 | 0 | AC |
| E29 | 57 | 23.42 | 14 | 2 | 0 | AC |
| E30 | 50 | 19.98 | 13 | 2 | 0 | AC |
| O01 | 50 | 25.68 | 14 | 2 | 1 | AC |
| O02 | 19 | 26.42 | 13 | 0 | 0 | AC |
| O03 | 51 | 29.82 | 13 | 1 | 0 | AC |
| O04 | 40 | 20.43 | 15 | 2 | 1 | AC |
| O05 | 45 | 23.16 | 15 | 1 | 0 | SCST |
| O06 | 45 | 19.55 | 15 | 2 | 3 | AC |
| O07 | 30 | 26.56 | 14 | 2 | 1 | SCST |
| O08 | 50 | 27.96 | 15 | 1 | 2 | AC |
| O09 | 58 | 19.43 | 12 | 1 | 1 | AC |
| O10 | 58 | 23.83 | 14 | 2 | 0 | AC |
| O11 | 59 | 22.6 | 12 | 2 | 2 | AC |
| O12 | 37 | 25.07 | 11 | 0 | 0 | CCC |
| O13 | 55 | 30.06 | 14 | 3 | 0 | AC |
| O14 | 56 | 23.14 | 15 | 2 | 0 | AC |
| O15 | 70 | 29.43 | 14 | 2 | 0 | SpCC |
| O16 | 49 | 21.1 | 14 | 3 | 1 | AC |
| O17 | 53 | 24.56 | 14 | 1 | 1 | AC |
| O18 | 65 | 27.16 | 13 | 8 | 0 | AC |
| O19 | 38 | 18.67 | 13 | 2 | 0 | AC |
| O20 | 66 | 24.24 | 13 | 3 | 0 | AC |
| O21 | 42 | 19.22 | 15 | 2 | 3 | AC |
| O22 | 56 | 20.5 | 13 | 3 | 0 | AC |
| O23 | 55 | 20 | 13 | 3 | 0 | AC |
| O24 | 61 | 21.45 | 12 | 2 | 1 | AC |
| O25 | 71 | 19.56 | 14 | 3 | 0 | AC |
| O26 | 39 | 21.27 | 13 | 3 | 0 | AC |
| O27 | 66 | 20.03 | 14 | 2 | 1 | SCST |
| O28 | 47 | 20.95 | 14 | 2 | 2 | AC |
| O29 | 64 | 17.93 | 13 | 3 | 1 | AC |
| O30 | 46 | 20.83 | 15 | 2 | 1 | AC |

SCC: Squamous cell carcinoma; AC: Adenocarcinoma; NEC: Neuroendocrine carcinoma; SCST: Sex cord-stromal tumor; CCC: Clear cell carcinoma; SpCC: Spindle cell carcinoma.

Hemogram

| **Patient**  **ID** | | **WBC**  **(×10^12^/mL)** | **RBC**  **(×10^12^/mL)** | **HGB**  **(g/mL)** | **PLT**  **(×10^9^/mL)** | **NEUT%**  **(%)** | **LYMPH%**  **(%)** | **NEUT #**  **(×10^5^/mL)** | **LYMPH#**  **(×10^9^/mL)** |
| --- | --- | --- | --- | --- | --- | --- | --- | --- | --- |
| C01 | 5.02 | | 4.15 | 91.00 | 411.00 | 53.70 | 34.30 | 2.70 | 1.72 |
| C02 | 3.82 | | 3.83 | 122.00 | 102.00 | 63.60 | 29.60 | 2.43 | 1.13 |
| C03 | 5.98 | | 4.48 | 127.00 | 219.00 | 60.20 | 29.40 | 3.60 | 1.76 |
| C04 | 3.83 | | 3.88 | 116.00 | 185.00 | 38.10 | 44.10 | 1.46 | 1.69 |
| C05 | 4.26 | | 3.78 | 120.00 | 201.00 | 70.90 | 17.10 | 3.02 | 0.73 |
| C06 | 4.45 | | 4.01 | 118.00 | 234.00 | 51.30 | 38.40 | 2.28 | 1.71 |
| C07 | 1.95 | | 3.33 | 107.00 | 264.00 | 44.20 | 41.60 | 0.87 | 0.81 |
| C08 | 10.31 | | 4.42 | 133.00 | 211.00 | 71.60 | 17.20 | 7.38 | 1.77 |
| C09 | 9.30 | | 4.21 | 128.00 | 261.00 | 69.20 | 16.20 | 6.43 | 1.51 |
| C10 | 9.54 | | 4.46 | 143.00 | 208.00 | 69.00 | 23.40 | 6.58 | 2.23 |
| C11 | 6.39 | | 4.37 | 125.00 | 302.00 | 67.20 | 22.20 | 4.29 | 1.42 |
| C12 | 4.63 | | 3.67 | 115.00 | 256.00 | 61.30 | 30.80 | 2.83 | 1.43 |
| C13 | 3.41 | | 4.04 | 101.00 | 241.00 | 53.70 | 37.30 | 1.83 | 1.26 |
| C14 | 4.86 | | 4.09 | 127.00 | 220.00 | 63.20 | 27.20 | 3.07 | 1.32 |
| C15 | 4.83 | | 4.71 | 117.00 | 265.00 | 69.70 | 28.00 | 3.37 | 1.35 |
| C16 | 5.11 | | 4.56 | 137.00 | 165.00 | 64.80 | 30.70 | 3.31 | 30.70 |
| C17 | 6.53 | | 3.94 | 120.00 | 270.00 | 54.70 | 34.30 | 3.57 | 2.24 |
| C18 | 5.49 | | 4.11 | 89.00 | 302.00 | 66.80 | 24.60 | 3.67 | 1.35 |
| C19 | 4.25 | | 4.15 | 75.00 | 300.00 | 59.10 | 35.80 | 2.51 | 1.53 |
| C20 | 4.87 | | 4.23 | 125.00 | 282.00 | 46.80 | 43.50 | 2.28 | 2.12 |
| C21 | 5.49 | | 3.68 | 116.00 | 257.00 | 65.60 | 24.60 | 3.60 | 1.35 |
| C22 | 8.18 | | 4.43 | 136.00 | 271.00 | 63.90 | 23.20 | 5.22 | 0.54 |
| C23 | 9.56 | | 3.95 | 127.00 | 266.00 | 77.40 | 16.10 | 7.40 | 1.54 |
| C24 | 4.48 | | 3.53 | 113.00 | 243.00 | 56.30 | 34.30 | 2.52 | 1.53 |
| C25 | 4.22 | | 4.09 | 127.00 | 239.00 | 43.50 | 45.30 | 1.84 | 1.91 |
| C26 | 5.38 | | 4.26 | 124.00 | 247.00 | 66.10 | 30.30 | 3.56 | 1.63 |
| C27 | 8.74 | | 4.32 | 130.00 | 318.00 | 66.50 | 26.30 | 5.81 | 2.30 |
| C28 | 4.96 | | 3.87 | 123.00 | 218.00 | 55.00 | 37.10 | 2.73 | 1.84 |
| C29 | 3.88 | | 3.87 | 122.00 | 218.00 | 53.10 | 36.60 | 2.06 | 1.42 |
| C30 | 7.24 | | 3.49 | 94.00 | 156.00 | 86.90 | 9.00 | 6.30 | 0.65 |
| E01 | 6.48 | | 5.09 | 145.00 | 330.00 | 58.60 | 32.10 | 2.08 | 0.29 |
| E02 | 12.78 | | 3.79 | 119.00 | 212.00 | 83.40 | 10.90 | 10.67 | 1.39 |
| E03 | 4.87 | | 4.11 | 128.00 | 222.00 | 53.20 | 37.80 | 2.59 | 1.84 |
| E04 | 9.69 | | 4.99 | 154.00 | 220.00 | 79.70 | 18.20 | 7.73 | 1.76 |
| E05 | 6.63 | | 4.34 | 132.00 | 267.00 | 56.00 | 31.80 | 3.72 | 2.11 |
| E06 | 4.28 | | 3.92 | 115.00 | 383.00 | 55.30 | 34.20 | 2.37 | 1.46 |
| E07 | 4.22 | | 4.72 | 100.00 | 296.00 | 65.50 | 24.90 | 2.76 | 1.05 |
| E08 | 4.80 | | 5.33 | 111.00 | 201.00 | 68.50 | 22.40 | 3.30 | 1.07 |
| E09 | 6.00 | | 4.44 | 133.00 | 223.00 | 64.10 | 25.00 | 3.85 | 1.50 |
| E10 | 7.06 | | 4.53 | 138.00 | 201.00 | 53.50 | 41.50 | 3.78 | 2.93 |
| E11 | 4.67 | | 4.20 | 122.00 | 175.00 | 64.90 | 24.80 | 3.03 | 1.16 |
| E12 | 8.00 | | 3.92 | 112.00 | 303.00 | 73.50 | 17.80 | 5.89 | 1.42 |
| E13 | 17.16 | | 4.00 | 112.00 | 211.00 | 88.70 | 6.10 | 15.20 | 1.04 |
| E14 | 3.69 | | 4.38 | 135.00 | 219.00 | 59.60 | 31.50 | 2.21 | 1.16 |
| E15 | 5.34 | | 4.62 | 103.00 | 386.00 | 73.30 | 19.40 | 3.92 | 1.03 |
| E16 | 5.63 | | 4.17 | 125.00 | 270.00 | 60.20 | 28.60 | 3.39 | 1.61 |
| E17 | 4.31 | | 3.66 | 112.00 | 202.00 | 69.90 | 25.90 | 3.00 | 1.12 |
| E18 | 6.74 | | 4.34 | 132.00 | 281.00 | 76.30 | 18.50 | 5.14 | 1.25 |
| E19 | 4.70 | | 3.92 | 132.00 | 243.00 | 50.90 | 38.30 | 2.39 | 1.80 |
| E20 | 5.70 | | 3.73 | 118.00 | 264.00 | 69.00 | 24.00 | 3.93 | 1.73 |
| E21 | 11.53 | | 4.40 | 129.00 | 190.00 | 79.10 | 14.20 | 9.12 | 1.64 |
| E22 | 4.88 | | 4.05 | 86.00 | 333.00 | 60.20 | 6.20 | 2.94 | 1.59 |
| E23 | 9.45 | | 4.70 | 148.00 | 274.00 | 81.90 | 14.50 | 7.74 | 1.37 |
| E24 | 6.45 | | 3.54 | 106.00 | 236.00 | 62.10 | 28.10 | 4.01 | 1.81 |
| E25 | 7.20 | | 3.83 | 74.00 | 349.00 | 42.30 | 48.60 | 3.05 | 3.50 |
| E26 | 4.80 | | 3.83 | 114.00 | 264.00 | 53.80 | 36.40 | 2.58 | 1.75 |
| E27 | 5.84 | | 3.55 | 89.00 | 430.00 | 63.00 | 23.80 | 3.68 | 1.39 |
| E28 | 4.76 | | 3.98 | 117.00 | 368.00 | 56.50 | 29.10 | 2.69 | 1.38 |
| E29 | 4.25 | | 3.97 | 125.00 | 172.00 | 57.40 | 30.90 | 2.45 | 1.31 |
| E30 | 7.40 | | 3.77 | 110.00 | 306.00 | 73.20 | 17.00 | 5.41 | 1.26 |
| O01 | 4.12 | | 4.03 | 134.00 | 197.00 | 63.00 | 23.90 | 2.61 | 0.98 |
| O02 | 8.49 | | 4.89 | 98.00 | 332.00 | 64.70 | 26.70 | 5.48 | 2.26 |
| O03 | 5.64 | | 4.68 | 138.00 | 181.00 | 58.20 | 32.90 | 3.28 | 1.86 |
| O04 | 4.13 | | 2.83 | 81.00 | 223.00 | 69.80 | 22.00 | 2.88 | 0.91 |
| O05 | 6.53 | | 5.22 | 138.00 | 242.00 | 76.60 | 17.70 | 5.00 | 1.16 |
| O06 | 3.93 | | 4.02 | 86.00 | 358.00 | 51.60 | 32.00 | 2.03 | 1.26 |
| O07 | 4.92 | | 4.29 | 124.00 | 144.00 | 66.30 | 26.80 | 3.26 | 26.80 |
| O08 | 5.60 | | 2.45 | 72.00 | 263.00 | 78.30 | 13.20 | 4.38 | 0.74 |
| O09 | 5.02 | | 4.15 | 91.00 | 411.00 | 53.70 | 34.30 | 2.70 | 1.72 |
| O10 | 3.82 | | 3.83 | 122.00 | 102.00 | 63.60 | 29.60 | 2.43 | 1.13 |
| O11 | 5.98 | | 4.48 | 127.00 | 219.00 | 60.20 | 29.40 | 3.60 | 1.76 |
| O12 | 3.83 | | 3.88 | 116.00 | 185.00 | 38.10 | 44.10 | 1.46 | 1.69 |
| O13 | 4.26 | | 3.78 | 120.00 | 201.00 | 70.90 | 17.10 | 3.02 | 0.73 |
| O14 | 4.45 | | 4.01 | 118.00 | 234.00 | 51.30 | 38.40 | 2.28 | 1.71 |
| O15 | 1.95 | | 3.33 | 107.00 | 264.00 | 44.20 | 41.60 | 0.87 | 0.81 |
| O16 | 10.31 | | 4.42 | 133.00 | 211.00 | 71.60 | 17.20 | 7.38 | 1.77 |
| O17 | 9.30 | | 4.21 | 128.00 | 261.00 | 69.20 | 16.20 | 6.43 | 1.51 |
| O18 | 9.54 | | 4.46 | 143.00 | 208.00 | 69.00 | 23.40 | 6.58 | 2.23 |
| O19 | 6.39 | | 4.37 | 125.00 | 302.00 | 67.20 | 22.20 | 4.29 | 1.42 |
| O20 | 4.63 | | 3.67 | 115.00 | 256.00 | 61.30 | 30.80 | 2.83 | 1.43 |
| O21 | 3.41 | | 4.04 | 101.00 | 241.00 | 53.70 | 37.30 | 1.83 | 1.26 |
| O22 | 4.86 | | 4.09 | 127.00 | 220.00 | 63.20 | 27.20 | 3.07 | 1.32 |
| O23 | 4.83 | | 4.71 | 117.00 | 265.00 | 69.70 | 28.00 | 3.37 | 1.35 |
| O24 | 5.11 | | 4.56 | 137.00 | 165.00 | 64.80 | 30.70 | 3.31 | 30.70 |
| O25 | 6.53 | | 3.94 | 120.00 | 270.00 | 54.70 | 34.30 | 3.57 | 2.24 |
| O26 | 5.49 | | 4.11 | 89.00 | 302.00 | 66.80 | 24.60 | 3.67 | 1.35 |
| O27 | 4.25 | | 4.15 | 75.00 | 300.00 | 59.10 | 35.80 | 2.51 | 1.53 |
| O28 | 4.87 | | 4.23 | 125.00 | 282.00 | 46.80 | 43.50 | 2.28 | 2.12 |
| O29 | 5.49 | | 3.68 | 116.00 | 257.00 | 65.60 | 24.60 | 3.60 | 1.35 |
| O30 | 8.18 | | 4.43 | 136.00 | 271.00 | 63.90 | 23.20 | 5.22 | 0.54 |

RBC: Red blood cell; WBC: White blood cell; HGB: Haemoglobin; PLT: Platelet; NEUT#: Neutrophil number; NEUT%: Neutrophil ratio; LYMPH#: Lymphocyte number; LYMPH%: Lymphocyte ratio.

Blood Biochemistry

| **Patient ID** | **TP**  **(g/L)** | **Albumin**  **(g/mL)** | **TBil**  **(μmol/L)** | **AST**  **(U/L)** | **ALT**  **(U/L)** | **Cre**  **(μmol/L)** | **UA**  **(μmol/L)** |
| --- | --- | --- | --- | --- | --- | --- | --- |
| C01 | 65.50 | 40.50 | 6.10 | 14.70 | 10.00 | 62.20 | 147.90 |
| C02 | 70.50 | 42.60 | 21.20 | 17.50 | 14.70 | 74.30 | 206.50 |
| C03 | 73.30 | 46.30 | 13.40 | 18.30 | 13.10 | 52.30 | 306.20 |
| C04 | 68.53 | 38.14 | 10.62 | 24.34 | 18.00 | 65.39 | 278.32 |
| C05 | 64.80 | 39.95 | 10.03 | 53.41 | 83.34 | 48.12 | 239.77 |
| C06 | 67.30 | 38.98 | 7.69 | 17.39 | 7.89 | 54.34 | 257.81 |
| C07 | 80.90 | 50.00 | 14.70 | 28.00 | 24.00 | 77.00 | 206.00 |
| C08 | 68.90 | 43.30 | 6.70 | 17.00 | 17.10 | 58.70 | 377.40 |
| C09 | 72.70 | 38.90 | 7.40 | 15.00 | 10.00 | 67.00 | 210.00 |
| C10 | 75.31 | 47.11 | 6.77 | 22.41 | 22.96 | 61.47 | 267.87 |
| C11 | 72.77 | 45.07 | 6.35 | 19.30 | 11.10 | 44.53 | 266.74 |
| C12 | 66.60 | 38.80 | 6.40 | 17.00 | 11.00 | 59.00 | 374.00 |
| C13 | 60.20 | 39.70 | 11.30 | 15.00 | 12.00 | 62.00 | 227.00 |
| C14 | 71.90 | 46.60 | 14.90 | 19.20 | 10.10 | 80.90 | 331.30 |
| C15 | 71.37 | 45.58 | 7.94 | 20.42 | 19.71 | 35.08 | 308.82 |
| C16 | 68.69 | 42.01 | 10.06 | 27.41 | 21.36 | 51.51 | 298.83 |
| C17 | 80.23 | 37.50 | 8.16 | 38.75 | 19.17 | 70.80 | 307.78 |
| C18 | 72.10 | 44.30 | 7.20 | 19.00 | 12.00 | 42.00 | 344.00 |
| C19 | 68.48 | 38.40 | 9.23 | 17.21 | 11.79 | 52.14 | 225.15 |
| C20 | 75.70 | 46.30 | 9.40 | 26.30 | 42.60 | 59.90 | 284.10 |
| C21 | 67.05 | 41.57 | 2.56 | 17.45 | 9.49 | 66.42 | 252.03 |
| C22 | 66.30 | 42.10 | 5.00 | 13.60 | 10.20 | 56.20 | 268.70 |
| C23 | 78.50 | 48.20 | 9.70 | 16.20 | 13.50 | 56.70 | 442.90 |
| C24 | 68.20 | 40.30 | 9.20 | 14.50 | 13.40 | 52.70 | 280.20 |
| C25 | 63.66 | 40.87 | 8.86 | 18.26 | 12.00 | 79.21 | 227.19 |
| C26 | 76.51 | 45.28 | 9.64 | 20.84 | 21.03 | 49.48 | 230.00 |
| C27 | 72.93 | 42.43 | 9.43 | 16.50 | 10.50 | 51.91 | 221.41 |
| C28 | 73.90 | 44.56 | 14.09 | 19.44 | 14.00 | 68.09 | 207.03 |
| C29 | 71.60 | 41.80 | 13.50 | 28.20 | 40.70 | 52.90 | 396.80 |
| C30 | 75.10 | 44.60 | 12.40 | 15.00 | 24.00 | 65.00 | 205.00 |
| E01 | 79.71 | 49.68 | 12.22 | 27.31 | 27.64 | 77.89 | 416.41 |
| E02 | 51.90 | 30.50 | 16.00 | 15.40 | 9.30 | 68.40 | 175.10 |
| E03 | 72.80 | 44.26 | 22.32 | 20.05 | 10.08 | 65.59 | 278.72 |
| E04 | 76.71 | 40.82 | 12.43 | 31.68 | 48.68 | 78.52 | 397.79 |
| E05 | 72.80 | 41.20 | 9.90 | 17.00 | 11.00 | 71.00 | 342.00 |
| E06 | 73.00 | 35.80 | 9.70 | 12.00 | 11.00 | 67.00 | 235.00 |
| E07 | 72.50 | 40.00 | 8.80 | 13.00 | 10.00 | 36.00 | 187.00 |
| E08 | 66.60 | 37.70 | 7.70 | 22.00 | 20.00 | 49.00 | 243.00 |
| E09 | 72.30 | 41.26 | 6.77 | 25.22 | 15.80 | 67.71 | 341.11 |
| E10 | 72.60 | 43.20 | 13.00 | 75.00 | 116.00 | 56.00 | 382.00 |
| E11 | 65.76 | 37.19 | 17.81 | 39.06 | 33.88 | 52.37 | 242.96 |
| E12 | 58.50 | 36.30 | 1.90 | 17.40 | 12.20 | 50.70 | 280.30 |
| E13 | 49.80 | 31.90 | 16.50 | 22.00 | 25.00 | 39.00 | 194.00 |
| E14 | 65.90 | 42.90 | 8.40 | 28.00 | 25.00 | 52.00 | 292.00 |
| E15 | 75.40 | 45.90 | 18.00 | 16.00 | 10.30 | 63.00 | 244.00 |
| E16 | 67.40 | 40.00 | 9.30 | 17.00 | 17.00 | 89.00 | 337.00 |
| E17 | 72.50 | 46.20 | 17.40 | 29.00 | 35.00 | 51.00 | 250.00 |
| E18 | 79.00 | 45.78 | 9.25 | 22.36 | 8.61 | 60.73 | 277.53 |
| E19 | 72.50 | 48.00 | 8.10 | 26.60 | 24.90 | 73.70 | 326.90 |
| E20 | 67.64 | 39.70 | 10.62 | 18.26 | 18.58 | 62.12 | 338.82 |
| E21 | 65.40 | 41.40 | 8.00 | 17.20 | 25.40 | 72.00 | 265.00 |
| E22 | 72.48 | 43.47 | 6.21 | 15.62 | 9.88 | 57.14 | 211.22 |
| E23 | 75.00 | 49.70 | 9.80 | 30.00 | 51.00 | 60.50 | 461.90 |
| E24 | 72.40 | 45.80 | 12.30 | 18.40 | 18.60 | 64.40 | 278.20 |
| E25 | 74.96 | 42.84 | 6.79 | 75.57 | 83.74 | 51.86 | 379.78 |
| E26 | 66.80 | 40.70 | 3.23 | 15.70 | 9.50 | 59.70 | 216.50 |
| E27 | 66.90 | 28.58 | 7.57 | 17.30 | 12.90 | 50.54 | 142.02 |
| E28 | 75.80 | 38.70 | 6.60 | 118.00 | 112.00 | 109.00 | 826.00 |
| E29 | 79.10 | 47.40 | 18.60 | 25.00 | 19.00 | 84.00 | 357.00 |
| E30 | 67.54 | 34.69 | 18.16 | 11.64 | 9.57 | 39.48 | 137.32 |
| O01 | 70.30 | 44.60 | 23.20 | 39.00 | 35.00 | 70.00 | 460.00 |
| O02 | 64.24 | 39.79 | 11.20 | 19.52 | 13.56 | 4.34 | 392.97 |
| O03 | 67.70 | 42.90 | 15.50 | 23.00 | 28.00 | 57.00 | 352.00 |
| O04 | 72.30 | 34.80 | 7.90 | 13.00 | 10.00 | 59.00 | 265.00 |
| O05 | 77.30 | 48.40 | 17.20 | 29.00 | 27.00 | 43.00 | 329.00 |
| O06 | 70.70 | 37.80 | 8.20 | 115.00 | 115.00 | 54.00 | 238.00 |
| O07 | 65.10 | 41.80 | 12.38 | 15.31 | 12.71 | 49.79 | 217.08 |
| O08 | 62.80 | 35.10 | 7.10 | 7.00 | 6.00 | 46.00 | 142.00 |
| O09 | 59.81 | 35.60 | 11.03 | 26.33 | 12.51 | 62.46 | 266.83 |
| O10 | 80.70 | 34.70 | 9.20 | 14.50 | 10.90 | 50.50 | 311.60 |
| O11 | 72.00 | 42.10 | 18.30 | 16.20 | 8.00 | 56.30 | 257.20 |
| O12 | 72.80 | 38.60 | 7.20 | 15.10 | 6.20 | 2.48 | 295.50 |
| O13 | 68.40 | 43.90 | 11.40 | 21.10 | 16.10 | 54.60 | 184.60 |
| O14 | 48.20 | 30.00 | 15.70 | 14.00 | 21.00 | 66.00 | 359.00 |
| O15 | 45.70 | 24.30 | 9.90 | 17.00 | 9.00 | 73.00 | 230.00 |
| O16 | 75.17 | 41.73 | 8.97 | 15.72 | 10.33 | 41.28 | 295.99 |
| O17 | 70.20 | 37.10 | 15.10 | 25.00 | 20.00 | 48.00 | 260.00 |
| O18 | 71.40 | 38.20 | 8.10 | 35.20 | 25.20 | 55.60 | 262.20 |
| O19 | 73.30 | 42.90 | 6.94 | 28.32 | 19.65 | 39.41 | 197.47 |
| O20 | 77.17 | 43.86 | 2.72 | 15.90 | 10.19 | 51.61 | 275.76 |
| O21 | 63.70 | 38.40 | 8.20 | 25.00 | 18.00 | 56.00 | 329.00 |
| O22 | 76.61 | 43.86 | 6.36 | 83.32 | 91.01 | 38.45 | 199.45 |
| O23 | 71.88 | 42.31 | 14.63 | 19.83 | 14.00 | 52.66 | 268.02 |
| O24 | 76.40 | 43.90 | 9.00 | 14.40 | 11.50 | 82.00 | 382.30 |
| O25 | 75.59 | 37.93 | 4.99 | 17.52 | 12.69 | 65.48 | 224.46 |
| O26 | 77.98 | 47.53 | 9.51 | 14.77 | 7.38 | 51.36 | 177.07 |
| O27 | 71.08 | 40.23 | 6.84 | 69.46 | 63.30 | 52.92 | 257.56 |
| O28 | 57.52 | 35.22 | 9.64 | 23.16 | 15.30 | 67.08 | 302.46 |
| O29 | 67.60 | 39.70 | 11.20 | 30.00 | 40.00 | 62.00 | 172.00 |
| O30 | 60.36 | 37.89 | 15.60 | 30.39 | 24.95 | 51.68 | 209.50 |

TP: Total protein; TBil: Total Bilirubin; AST: Aspartic transaminase; ALT: alanine aminotransferase; Cre: Creatinine; UA: Uric acid.

Supplementary information S2 DNA Extraction and Amplification

Tumoural DNA extraction

Instruments and reagents

| **Instruments/Reagents** | **Catalogue and Provider** |
| --- | --- |
| Microultraviolet spectrophotometer | NC2000, Thermo Scientific |
| Electrophoresis apparatus | DYY-6C, Beijing Liuyi Biotechnology |
| Gel imaging system | BG-gds AUTO (130), Baygen |
| Agarose | 75510-019, Invitrogen |
| DNA Marker | DL15,000, Takara |
| Tris-acetate-EDTA (TAE) buffer | AM9870, Invitrogen |

Ultraviolet spectrophotometry result A

| **Lane** | **Sample ID** | **Concentration** | **OD_260/280_** | **OD_260/230_** | **Total volume** | **DNA weight** |
| --- | --- | --- | --- | --- | --- | --- |
| 1 | C01 | 454.53 ng/μL | 1.844 | 0.451 | 50 μL | 22.726 μg |
| 2 | C02 | 1254.39 ng/μL | 1.835 | 0.443 | 50 μL | 62.720 μg |
| 3 | C03 | 211.90 ng/μL | 1.841 | 0.474 | 50 μL | 10.595 μg |
| 4 | C04 | 1424.16 ng/μL | 1.821 | 0.425 | 50 μL | 71.208 μg |
| 5 | C05 | 781.89 ng/μL | 1.819 | 0.634 | 50 μL | 39.095 μg |
| 6 | C06 | 408.19 ng/μL | 1.836 | 0.458 | 50 μL | 20.409 μg |
| 7 | C07 | 976.51 ng/μL | 1.841 | 0.583 | 50 μL | 48.825 μg |
| 8 | C08 | 596.66 ng/μL | 1.842 | 0.664 | 50 μL | 29.833 μg |
| 9 | C09 | 1584.65 ng/μL | 1.837 | 0.643 | 50 μL | 79.233 μg |
| 10 | C10 | 66.25 ng/μL | 1.771 | 0.651 | 50 μL | 3.313 μg |
| 11 | C11 | 190.29 ng/μL | 1.797 | 0.371 | 50 μL | 9.515 μg |
| 12 | C12 | 1098.41 ng/μL | 1.838 | 0.385 | 50 μL | 54.920 μg |
| 13 | C13 | 111.88 ng/μL | 1.738 | 0.436 | 50 μL | 5.594 μg |
| 14 | C14 | 421.02 ng/μL | 1.829 | 0.305 | 50 μL | 21.051 μg |
| 15 | C15 | 168.39 ng/μL | 1.756 | 0.364 | 50 μL | 8.419 μg |
| 16 | C16 | 837.18 ng/μL | 1.838 | 0.377 | 50 μL | 41.859 μg |
| 17 | C17 | 423.05 ng/μL | 1.833 | 0.341 | 50 μL | 21.152 μg |
| 18 | C18 | 1320.10 ng/μL | 1.835 | 0.366 | 50 μL | 66.005 μg |
| 19 | C19 | 167.25 ng/μL | 1.778 | 0.522 | 50 μL | 8.363 μg |
| 20 | C20 | 173.64 ng/μL | 1.819 | 0.445 | 50 μL | 8.682 μg |
| 21 | C21 | 495.27 ng/μL | 1.835 | 0.371 | 50 μL | 24.764 μg |
| 22 | C22 | 56.71 ng/μL | 1.882 | 0.422 | 50 μL | 2.836 μg |
| 23 | C23 | 59.51 ng/μL | 1.775 | 0.334 | 50 μL | 2.975 μg |
| 24 | C24 | 188.21 ng/μL | 1.811 | 0.365 | 50 μL | 9.411 μg |
| 25 | C25 | 178.78 ng/μL | 1.817 | 0.374 | 50 μL | 8.939 μg |
| 26 | O01 | 785.69 ng/μL | 1.821 | 0.433 | 50 μL | 39.284 μg |
| 27 | O02 | 699.22 ng/μL | 1.832 | 0.466 | 50 μL | 34.961 μg |
| 28 | O03 | 217.58 ng/μL | 1.808 | 0.477 | 50 μL | 10.879 μg |
| 29 | O04 | 1.85 ng/μL | 1.639 | 0.384 | 50 μL | 0.093 μg |
| 30 | O05 | 490.82 ng/μL | 1.796 | 0.364 | 50 μL | 24.541 μg |
| 31 | O06 | 1241.41 ng/μL | 1.817 | 0.388 | 50 μL | 62.070 μg |
| 32 | O07 | 255.26 ng/μL | 1.814 | 0.453 | 50 μL | 12.763 μg |
| 33 | O08 | 738.00 ng/μL | 1.805 | 0.421 | 50 μL | 36.900 μg |
| 34 | O09 | 505.84 ng/μL | 1.828 | 0.446 | 50 μL | 25.292 μg |
| 35 | O10 | 23.21 ng/μL | 1.812 | 0.362 | 50 μL | 1.161 μg |
| 36 | O11 | 818.06 ng/μL | 1.844 | 0.441 | 50 μL | 40.903 μg |
| 37 | O13 | 1149.92 ng/μL | 1.813 | 0.367 | 50 μL | 57.496 μg |
| 38 | O15 | 231.25 ng/μL | 1.819 | 0.521 | 50 μL | 11.562 μg |
| 39 | O16 | 661.54 ng/μL | 1.811 | 0.547 | 50 μL | 33.077 μg |
| 40 | O17 | 747.28 ng/μL | 1.819 | 0.436 | 50 μL | 37.364 μg |
| 41 | O18 | 161.82 ng/μL | 1.705 | 0.451 | 50 μL | 8.091 μg |
| 42 | O19 | 121.82 ng/μL | 1.788 | 0.447 | 50 μL | 6.091 μg |
| 43 | O20 | 441.47 ng/μL | 1.778 | 0.315 | 50 μL | 22.074 μg |
| 44 | O21 | 511.69 ng/μL | 1.813 | 0.346 | 50 μL | 25.585 μg |
| 45 | O22 | 369.06 ng/μL | 1.849 | 0.347 | 50 μL | 18.453 μg |
| 46 | O23 | 1128.23 ng/μL | 1.852 | 0.323 | 50 μL | 56.411 μg |
| 47 | O25 | 118.05 ng/μL | 1.828 | 0.691 | 50 μL | 5.902 μg |
| 48 | E01 | 1327.57 ng/μL | 1.856 | 0.671 | 50 μL | 66.379 μg |
| 49 | E02 | 51.02 ng/μL | 1.763 | 0.604 | 50 μL | 2.551 μg |
| 50 | E03 | 3055.13 ng/μL | 1.741 | 0.532 | 50 μL | 152.757 μg |
| 51 | E04 | 426.98 ng/μL | 1.803 | 0.522 | 50 μL | 21.349 μg |
| 52 | E05 | 598.86 ng/μL | 1.833 | 0.562 | 50 μL | 29.943 μg |
| 53 | E06 | 1345.39 ng/μL | 1.819 | 0.443 | 50 μL | 67.269 μg |
| 54 | E07 | 256.71 ng/μL | 1.762 | 0.435 | 50 μL | 12.835 μg |
| 55 | E08 | 287.33 ng/μL | 1.779 | 0.615 | 50 μL | 14.367 μg |
| 56 | E09 | 41.30 ng/μL | 1.764 | 0.435 | 50 μL | 2.065 μg |
| 57 | E10 | 1305.35 ng/μL | 1.815 | 0.431 | 50 μL | 65.267 μg |
| 58 | E11 | 2246.92 ng/μL | 1.832 | 0.336 | 50 μL | 112.346 μg |
| 59 | E12 | 56.45 ng/μL | 1.807 | 0.415 | 50 μL | 2.823 μg |
| 60 | E13 | 968.02 ng/μL | 1.813 | 0.345 | 50 μL | 48.401 μg |
| 61 | E14 | 34.11 ng/μL | 1.741 | 0.527 | 50 μL | 1.706 μg |
| 62 | E15 | 38.82 ng/μL | 1.804 | 0.345 | 50 μL | 1.941 μg |
| 63 | E16 | 44.28 ng/μL | 1.753 | 0.588 | 50 μL | 2.214 μg |
| 64 | E17 | 25.91 ng/μL | 1.788 | 0.541 | 50 μL | 1.296 μg |
| 65 | E18 | 204.51 ng/μL | 1.825 | 0.567 | 50 μL | 10.225 μg |
| 66 | E19 | 76.21 ng/μL | 1.832 | 0.476 | 50 μL | 3.811 μg |
| 67 | E20 | 163.00 ng/μL | 1.801 | 0.426 | 50 μL | 8.150 μg |
| 68 | E21 | 154.28 ng/μL | 1.829 | 0.554 | 50 μL | 7.714 μg |
| 69 | E22 | 64.16 ng/μL | 1.827 | 0.364 | 50 μL | 3.208 μg |
| 70 | E23 | 190.68 ng/μL | 1.682 | 0.371 | 50 μL | 9.534 μg |
| 71 | E24 | 214.10 ng/μL | 1.837 | 0.551 | 50 μL | 10.705 μg |
| 72 | E25 | 264.98 ng/μL | 1.841 | 0.635 | 50 μL | 13.249 μg |

Ultraviolet spectrophotometry result B

| **Lane** | **Sample ID** | **Concentration** | **OD_260/280_** | **OD_260/230_** | **Total volume** | **DNA weight** |
| --- | --- | --- | --- | --- | --- | --- |
| 1 | C26 | 191.85 ng/μL | 1.827 | 0.455 | 50 μL | 9.592 μg |
| 2 | C27 | 1013.45 ng/μL | 1.821 | 0.426 | 50 μL | 50.672 μg |
| 3 | C28 | 311.99 ng/μL | 1.801 | 0.477 | 50 μL | 15.600 μg |
| 4 | C29 | 636.81 ng/μL | 1.831 | 0.512 | 50 μL | 31.840 μg |
| 5 | C34 | 75.48 ng/μL | 1.663 | 0.536 | 50 μL | 3.774 μg |
| 6 | O26 | 5.46 ng/μL | 1.754 | 0.423 | 50 μL | 0.273 μg |
| 7 | O27 | 2.98 ng/μL | 1.725 | 0.443 | 50 μL | 0.149 μg |
| 8 | O28 | 373.55 ng/μL | 1.794 | 0.471 | 50 μL | 18.678 μg |
| 9 | O29 | 88.06 ng/μL | 1.758 | 0.528 | 50 μL | 4.403 μg |
| 10 | O30 | 33.64 ng/μL | 1.755 | 0.536 | 50 μL | 1.682 μg |
| 11 | O31 | 380.90 ng/μL | 1.805 | 0.544 | 50 μL | 19.045 μg |
| 12 | O33 | 108.30 ng/μL | 1.793 | 0.533 | 50 μL | 5.415 μg |
| 13 | O34 | 511.39 ng/μL | 1.832 | 0.361 | 50 μL | 25.570 μg |
| 14 | E26 | 471.19 ng/μL | 1.804 | 0.471 | 50 μL | 23.559 μg |
| 15 | E27 | 543.12 ng/μL | 1.768 | 0.446 | 50 μL | 27.156 μg |
| 16 | E28 | 195.61 ng/μL | 1.748 | 0.485 | 50 μL | 9.780 μg |
| 17 | E29 | 584.24 ng/μL | 1.779 | 0.435 | 50 μL | 29.212 μg |
| 18 | E32 | 389.02 ng/μL | 1.783 | 0.477 | 50 μL | 19.451 μg |

Agarose gel electrophoresis

Condition

| **Variables** | **Condition** |
| --- | --- |
| Marker | 5 μL |
| Sample | 5 μL |
| Time | 20 min |
| Agarose concentration | 1.2% |
| Voltage | 120 V (Constant) |
| Electric current | ~ 80mA |

Agarose gel electrophoresis blotting A


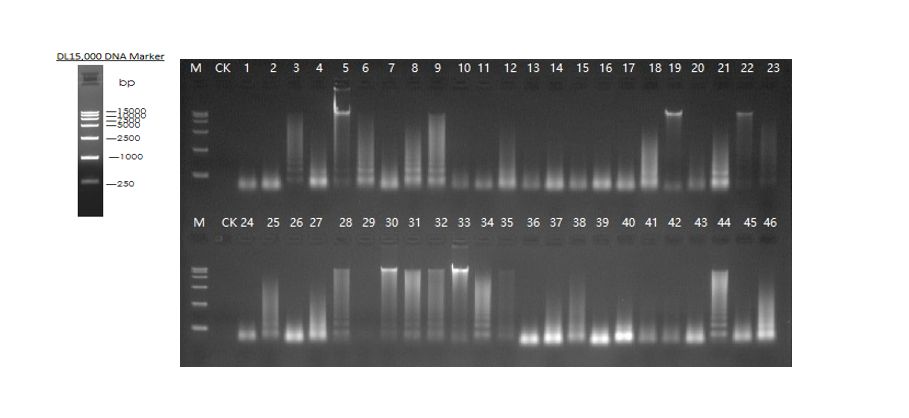


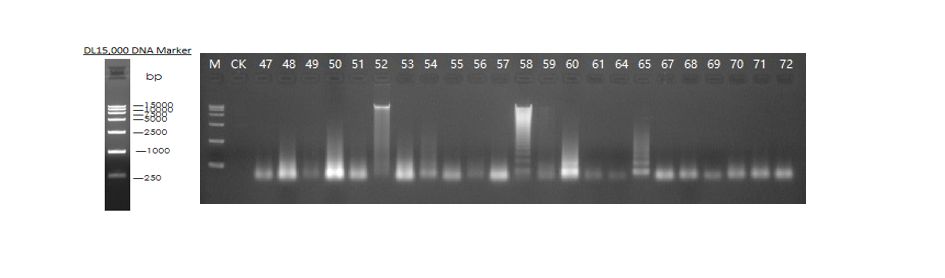


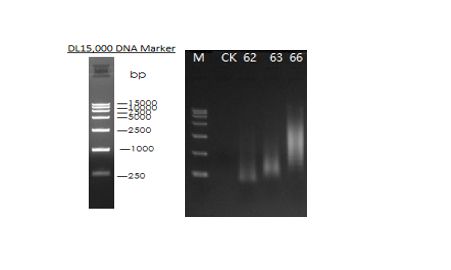


Agarose gel electrophoresis blotting B


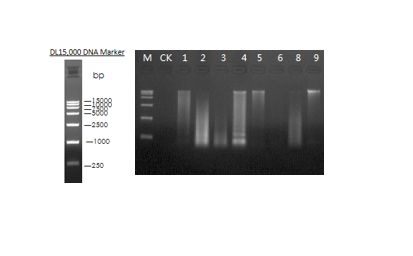


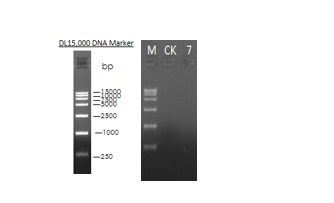


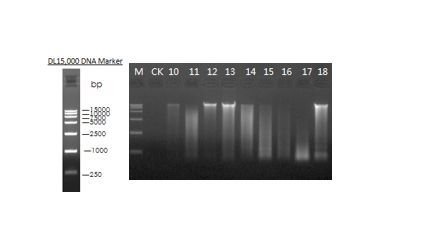


16S rDNA amplification

Instruments and reagents

| **Instruments/Reagents** | **Catalogue and Provider** |
| --- | --- |
| Polymerase chain reaction (PCR) amplifier | 2720, ABI |
| Microplate reader | FLX800T, BioTek |
| Electrophoresis apparatus | DYY-6C, Beijing Liuyi Biotechnology |
| Gel imaging system | BG-gds AUTO (130), Baygen |
| Q5® High-Fidelity DNA Polymerase | M0491L, NEB |
| Quant-iT PicoGreen dsDNA Assay Kit | P7589, Invitrogen |
| Agarose | 75510-019, Invitrogen |
| DNA Marker | DL2000, Takara |
| Tris-acetate-EDTA (TAE) buffer | AM9870, Invitrogen |

Primers

| **Primer** | **Sequence (5' end to 3' end)** | **Length (bp)** |
| --- | --- | --- |
| 27F | AGAGTTTGATCMTGGCTCAG | 20 |
| 1492R | ACCTTGTTACGACTT | 15 |

Amplification system

The sample is uniformly diluted to 20 ng/μL, and the insufficient sample is directly used in the stock solution. At the same time, the sample will be diluted to a certain extent according to the actual amplification of the sample.

The amplification system is constructed as follow:

| **Reagents** | **Volume** |
| --- | --- |
| 5 × reaction buffer | 5 μL |
| 5 × GC buffer | 5 μL |
| dNTP (2.5 mM) | 2 μL |
| Forward primer (10 μM) | 1 μL |
| Reverse primer (10 μM) | 1 μL |
| DNA Template | 2 μL |
| ddH_2_O | 8.75 μL |
| Q5 DNA Polymerase | 0.25 μL |
| Total | 25 μL |

The PCR amplification processes are set as follow:

| **Phase** | **Temperature** | **Time** |
| --- | --- | --- |
| Initial denaturation | 98 ℃ | 2 min |
| Denaturation | 98 ℃ | 15 s |
| Annealing | 55 ℃ | 30 s |
| Extension | 72 ℃ | 30 s |
| Final extension | 72 ℃ | 5 min |
| Hold | 10 ℃ | ∞ |
| Cycle | 25-30 | |

Amplification result A

PacBio Sequel II platform were used for sequencing.

| **Sample ID** | **Sample** | **Pre-primer ID** | **Pre-primer barcode** | **Length** | **Concentration** |
| --- | --- | --- | --- | --- | --- |
| ZTD22CB458 | C01 | 16S-PB296 | GACTCGCGATACTAGA | 1500 bp | 16.30 ng/μL |
| ZTD22CB459 | C02 | 16S-PB297 | TACTAGAGTAGCACTC | 1500 bp | 16.20 ng/μL |
| ZTD22CB460 | C03 | 16S-PB298 | CGAGTGTGTCTATACT | 1500 bp | 23.20 ng/μL |
| ZTD22CB461 | C04 | 16S-PB299 | ATCGCATCGCAGAGAC | 1500 bp | 18.20 ng/μL |
| ZTD22CB462 | C05 | 16S-PB300 | GTATAGACAGATGTGC | 1500 bp | 27.40 ng/μL |
| ZTD22CB463 | C06 | 16S-PB301 | GCGATCACTGTACACT | 1500 bp | 17.80 ng/μL |
| ZTD22CB464 | C07 | 16S-PB302 | ACAGTCTATACTGCTG | 1500 bp | 14.10 ng/μL |
| ZTD22CB465 | C08 | 16S-PB303 | ACTGTGACAGTATGAT | 1500 bp | 10.40 ng/μL |
| ZTD22CB466 | C09 | 16S-PB304 | ACGTGCTCTATAGAGA | 1500 bp | 16.60 ng/μL |
| ZTD22CB467 | C10 | 16S-PB305 | TAGTGTGCGACTCTGA | 1500 bp | 14.10 ng/μL |
| ZTD22CB468 | C11 | 16S-PB306 | TGCTATCTGAGATACT | 1500 bp | 6.22 ng/μL |
| ZTD22CB469 | C12 | 16S-PB307 | CAGATCTCGCGTGACA | 1500 bp | 6.00 ng/μL |
| ZTD22CB470 | C13 | 16S-PB308 | TCGCGACATATAGATG | 1500 bp | 14.90 ng/μL |
| ZTD22CB471 | C14 | 16S-PB309 | CTAGTCTCTATCGCAT | 1500 bp | 13.20 ng/μL |
| ZTD22CB472 | C15 | 16S-PB310 | CTACGAGACAGATCGC | 1500 bp | 15.10 ng/μL |
| ZTD22CB473 | C16 | 16S-PB311 | TGTCTGAGACGCATAC | 1500 bp | 13.70 ng/μL |
| ZTD22CB474 | C17 | 16S-PB312 | TGCGAGACTATCGCGA | 1500 bp | 16.10 ng/μL |
| ZTD22CB475 | C18 | 16S-PB313 | TATCAGCACGACATGC | 1500 bp | 13.80 ng/μL |
| ZTD22CB476 | C19 | 16S-PB314 | AGTCAGATGCGCACTC | 1500 bp | 18.70 ng/μL |
| ZTD22CB477 | C20 | 16S-PB315 | ACATGCGTGACAGTCA | 1500 bp | 15.30 ng/μL |
| ZTD22CB478 | C21 | 16S-PB356 | CTCGAGCAGTAGATAC | 1500 bp | 19.00 ng/μL |
| ZTD22CB479 | C22 | 16S-PB357 | GTATCGAGCGTATAGC | 1500 bp | 19.40 ng/μL |
| ZTD22CB480 | C23 | 16S-PB358 | GATAGCTGCTAGCTGA | 1500 bp | 21.00 ng/μL |
| ZTD22CB481 | C24 | 16S-PB359 | CGAGCTACTCTGACAG | 1500 bp | 13.10 ng/μL |
| ZTD22CB482 | C25 | 16S-PB360 | CGTATACAGTCACGCT | 1500 bp | 18.30 ng/μL |
| ZTD22CB483 | O01 | 16S-PB316 | AGCGTCTGACGTGAGT | 1500 bp | 14.60 ng/μL |
| ZTD22CB484 | O02 | 16S-PB317 | TGTGCACGACAGCAGT | 1500 bp | 13.50 ng/μL |
| ZTD22CB485 | O03 | 16S-PB318 | GACTCTCTATCGTACT | 1500 bp | 24.40 ng/μL |
| ZTD22CB486 | O04 | 16S-PB319 | ATGCTCACTACTACAT | 1500 bp | 20.40 ng/μL |
| ZTD22CB487 | O05 | 16S-PB320 | CAGCAGATCATGTCGA | 1500 bp | 17.40 ng/μL |
| ZTD22CB488 | O06 | 16S-PB321 | GATCATGTGAGCATAG | 1500 bp | 9.00 ng/μL |
| ZTD22CB489 | O07 | 16S-PB322 | CATCGTCTAGCACTCG | 1500 bp | 22.80 ng/μL |
| ZTD22CB490 | O08 | 16S-PB323 | GCACAGCGTAGCGCAT | 1500 bp | 23.20 ng/μL |
| ZTD22CB491 | O09 | 16S-PB324 | GACATAGCTAGATCGC | 1500 bp | 16.50 ng/μL |
| ZTD22CB492 | O10 | 16S-PB325 | CGTGAGTATATGTCAT | 1500 bp | 21.20 ng/μL |
| ZTD22CB493 | O11 | 16S-PB326 | CGACTAGATCTATCAT | 1500 bp | 15.20 ng/μL |
| ZTD22CB494 | O13 | 16S-PB327 | TACGCGTGTACGCAGA | 1500 bp | 15.60 ng/μL |
| ZTD22CB495 | O15 | 16S-PB328 | TGCGAGCGACTCTATC | 1500 bp | 18.80 ng/μL |
| ZTD22CB496 | O16 | 16S-PB329 | ACGCTCGCTGAGCATA | 1500 bp | 13.70 ng/μL |
| ZTD22CB497 | O17 | 16S-PB330 | TCACGTGCAGATATAG | 1500 bp | 11.40 ng/μL |
| ZTD22CB498 | O18 | 16S-PB331 | CATAGCGACTATCGTG | 1500 bp | 14.50 ng/μL |
| ZTD22CB499 | O19 | 16S-PB332 | CATATGCTCGTGCACT | 1500 bp | 16.60 ng/μL |
| ZTD22CB500 | O20 | 16S-PB333 | CGTACGTGCGAGTACA | 1500 bp | 15.20 ng/μL |
| ZTD22CB501 | O21 | 16S-PB334 | CGACGTCATAGTGCGT | 1500 bp | 21.80 ng/μL |
| ZTD22CB502 | O22 | 16S-PB335 | CGCTAGAGATCTGCTA | 1500 bp | 22.00 ng/μL |
| ZTD22CB503 | O23 | 16S-PB361 | ATCGAGCAGCAGTCGT | 1500 bp | 13.40 ng/μL |
| ZTD22CB504 | O25 | 16S-PB362 | GCTAGTCGATGACAGC | 1500 bp | 26.60 ng/μL |
| ZTD22CB505 | E01 | 16S-PB336 | CTGTAGACATCACACG | 1500 bp | 13.40 ng/μL |
| ZTD22CB506 | E02 | 16S-PB337 | AGCTACGCGTGCACTG | 1500 bp | 4.76 ng/μL |
| ZTD22CB507 | E03 | 16S-PB338 | CATATCAGTGCTACAG | 1500 bp | 8.32 ng/μL |
| ZTD22CB508 | E04 | 16S-PB339 | TAGAGCGTCTCTCGTA | 1500 bp | 2.54 ng/μL |
| ZTD22CB509 | E05 | 16S-PB340 | ATCGCTGTGTCTATAG | 1500 bp | 25.40 ng/μL |
| ZTD22CB510 | E06 | 16S-PB341 | ATGCTGATGACGCGCT | 1500 bp | 14.90 ng/μL |
| ZTD22CB511 | E07 | 16S-PB342 | CTGCGCAGTACGTGCA | 1500 bp | 16.10 ng/μL |
| ZTD22CB512 | E08 | 16S-PB343 | AGTGCGCACATGTCAG | 1500 bp | 13.10 ng/μL |
| ZTD22CB513 | E09 | 16S-PB344 | CGTGAGTAGTCAGACG | 1500 bp | 2.72 ng/μL |
| ZTD22CB514 | E10 | 16S-PB345 | GATCACAGAGATGCTC | 1500 bp | 11.40 ng/μL |
| ZTD22CB515 | E11 | 16S-PB346 | TCGAGTGTATAGCTCA | 1500 bp | 22.00 ng/μL |
| ZTD22CB516 | E12 | 16S-PB347 | GAGTCTGCACGCGCTA | 1500 bp | 18.00 ng/μL |
| ZTD22CB517 | E13 | 16S-PB348 | CGACTACGTACAGTAG | 1500 bp | 14.70 ng/μL |
| ZTD22CB518 | E14 | 16S-PB349 | AGCGTAGCATCTGAGC | 1500 bp | 9.04 ng/μL |
| ZTD22CB519 | E15 | 16S-PB350 | TACGATCGTAGCTGCT | 1500 bp | 12.50 ng/μL |
| ZTD22CB520 | E16 | 16S-PB351 | TCGCGAGCAGCGACAT | 1500 bp | 18.70 ng/μL |
| ZTD22CB521 | E17 | 16S-PB352 | ACTATCGCAGCTCAGT | 1500 bp | 16.20 ng/μL |
| ZTD22CB522 | E18 | 16S-PB353 | CTGATGCGCGCTGTAC | 1500 bp | 13.20 ng/μL |
| ZTD22CB523 | E19 | 16S-PB354 | CGTACGACTGCAGCGT | 1500 bp | 4.98 ng/μL |
| ZTD22CB524 | E20 | 16S-PB355 | ATCTGAGTCTGACACG | 1500 bp | 25.40 ng/μL |
| ZTD22CB525 | E21 | 16S-PB363 | GTACACGCTGTGACTA | 1500 bp | 15.80 ng/μL |
| ZTD22CB526 | E22 | 16S-PB364 | ACGTGTATGACGATAC | 1500 bp | 16.20 ng/μL |
| ZTD22CB527 | E23 | 16S-PB365 | CGTGCACATCTATAGC | 1500 bp | 13.20 ng/μL |
| ZTD22CB528 | E24 | 16S-PB366 | ACGATCACTCGTGTCA | 1500 bp | 21.20 ng/μL |
| ZTD22CB529 | E25 | 16S-PB367 | GCTCTCACGATATCAG | 1500 bp | 16.30 ng/μL |

Amplification result B

PacBio Sequel II platform were used for sequencing.

| **Sample ID** | **Sample** | **Pre-primer ID** | **Pre-primer barcode** | **Length** | **Concentration** |
| --- | --- | --- | --- | --- | --- |
| PSN22KU130 | C26 | 16S-PB368 | AGTCGCGTAGCTCATC | 1500 bp | 24.20 ng/μL |
| PSN22KU131 | C27 | 16S-PB369 | AGTCACTGTCTACTCG | 1500 bp | 24.40 ng/μL |
| PSN22KU132 | C28 | 16S-PB370 | TGACTCGCTCATAGTC | 1500 bp | 4.10 ng/μL |
| PSN22KU133 | C29 | 16S-PB371 | GTAGTCTCGCACAGAT | 1500 bp | 12.30 ng/μL |
| PSN22KU134 | C34 | 16S-PB372 | GATCGACTCGAGCATC | 1500 bp | 23.00 ng/μL |
| PSN22KU135 | O26 | 16S-PB373 | GATCTCTACTATATGC | 1500 bp | 19.80 ng/μL |
| PSN22KU136 | O27 | 16S-PB374 | CATGATGCGAGACGCT | 1500 bp | 17.90 ng/μL |
| PSN22KU137 | O28 | 16S-PB375 | AGCTCTGTCACTAGAC | 1500 bp | 19.80 ng/μL |
| PSN22KU138 | O29 | 16S-PB376 | CAGTCTGTGAGTCACT | 1500 bp | 22.40 ng/μL |
| PSN22KU139 | O30 | 16S-PB377 | TCGATATACGACGTGC | 1500 bp | 19.00 ng/μL |
| PSN22KU140 | O31 | 16S-PB378 | TCGATACGCACTCGAT | 1500 bp | 19.20 ng/μL |
| PSN22KU141 | O33 | 16S-PB379 | TCAGACGATGCGTCAT | 1500 bp | 23.40 ng/μL |
| PSN22KU142 | O34 | 16S-PB380 | GTCACGATATAGTGAC | 1500 bp | 15.00 ng/μL |
| PSN22KU143 | E26 | 16S-PB381 | ACTACATACTAGATCA | 1500 bp | 16.20 ng/μL |
| PSN22KU144 | E27 | 16S-PB382 | GAGAGTGTGAGAGTGT | 1500 bp | 15.80 ng/μL |
| PSN22KU145 | E28 | 16S-PB383 | ACTCAGCGCGTACATA | 1500 bp | 17.80 ng/μL |
| PSN22KU146 | E29 | 16S-PB384 | CGAGCACGCGCGTGTG | 1500 bp | 11.80 ng/μL |
| PSN22KU147 | E32 | 16S-PB001 | CACATATCAGAGTGCG | 1500 bp | 19.00 ng/μL |

Supplementary information S3 Functional Prediction of Metabolic Activity

Overall abundance of metabolic pathway

Cervical cancer (*n* = 30)


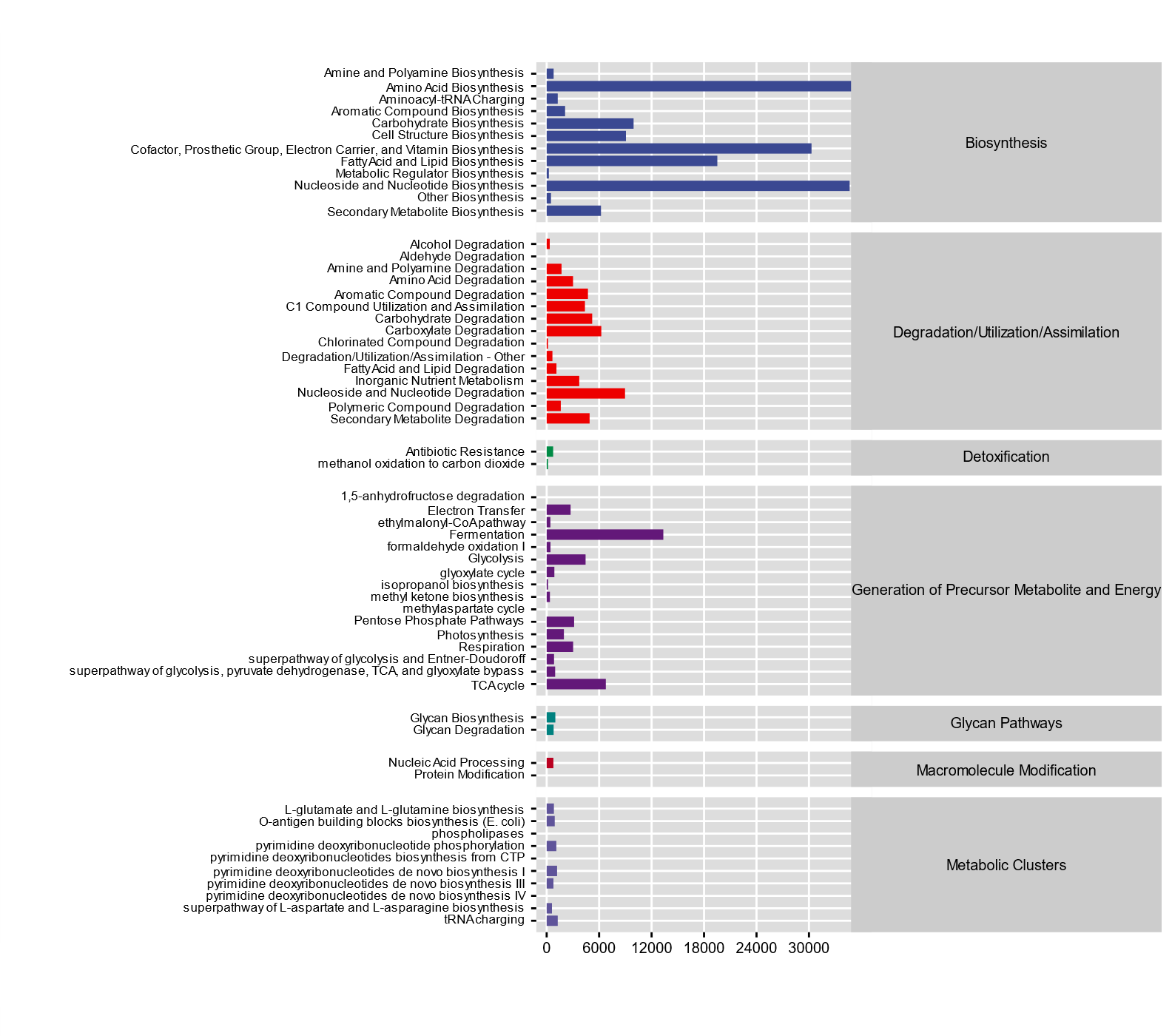


Ovarian cancer (*n* = 30)


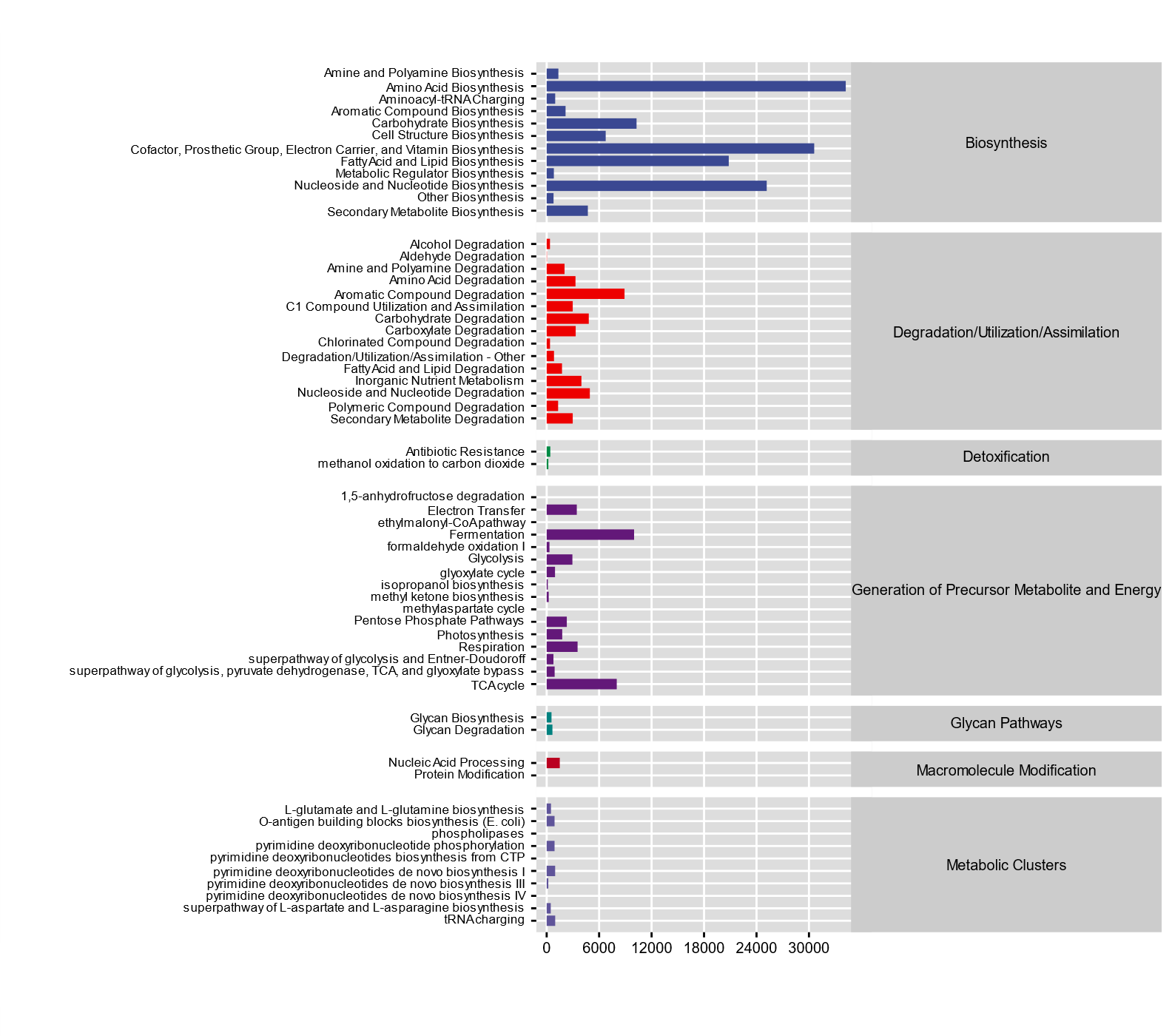


Endometrial cancer (*n* = 30)


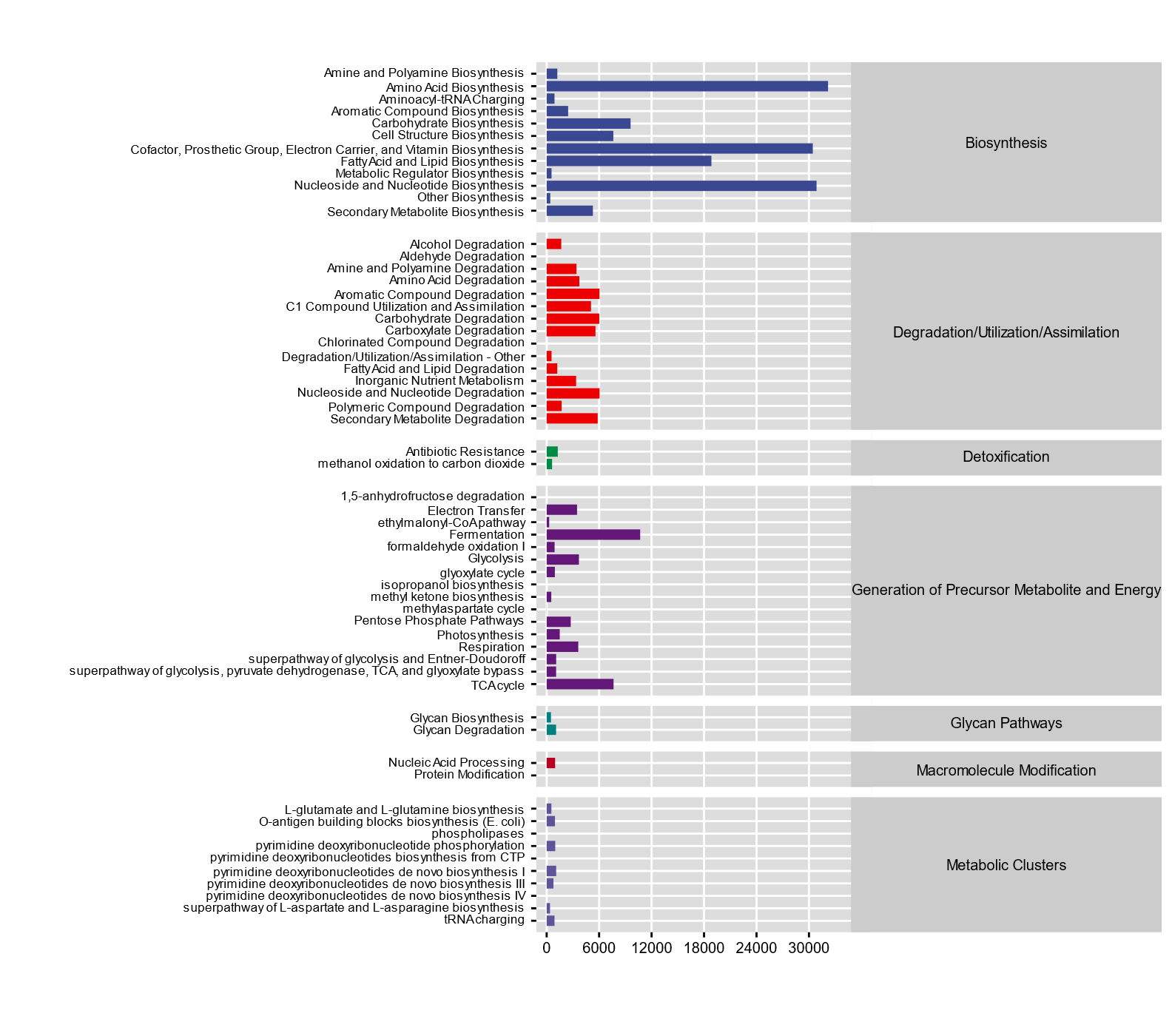


PCoA analysis of functional units


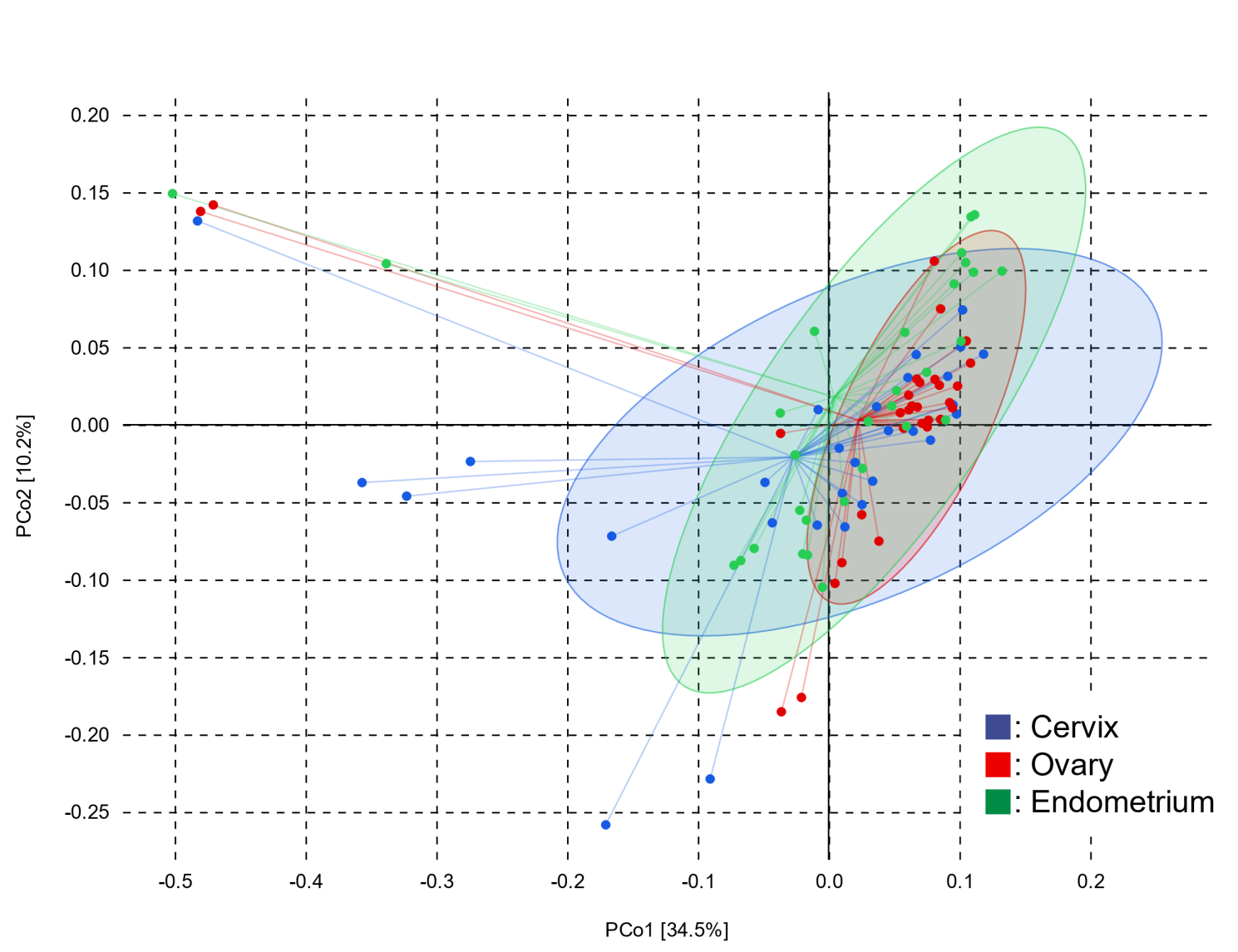


Supplementary information S4 Phylogenetic Analysis
